# Genes Associated With Psychrotolerant *Bacillus cereus* Group Isolates

**DOI:** 10.3389/fmicb.2019.00662

**Published:** 2019-03-29

**Authors:** Sarah M. Beno, Renato H. Orsi, Rachel A. Cheng, David J. Kent, Jasna Kovac, Diana R. Duncan, Nicole H. Martin, Martin Wiedmann

**Affiliations:** ^1^Department of Food Science, Cornell University, Ithaca, NY, United States; ^2^Department of Food Science, Penn State University, University Park, PA, United States; ^3^Department of Food Science, Wageningen University, Wageningen, Netherlands

**Keywords:** *Bacillus cereus*, whole genome sequencing, psychrotolerant, spoilage, skim milk broth

## Abstract

The *Bacillus cereus* group comprises 18 different species, including human pathogens as well as psychrotolerant strains that are an important cause of fluid milk spoilage. To enhance our understanding of the genetic markers associated with psychrotolerance (defined here as > 1 log_10_ increase in cfu/mL after 21 days incubation at 6°C) among dairy-associated *B. cereus* group isolates, we used genetic (whole genome sequencing) and phenotypic methods [growth in Skim Milk Broth (SMB) and Brain Heart Infusion (BHI) broth] to characterize 23 genetically-distinct representative isolates from a collection of 503 dairy-associated isolates. Quality threshold clustering identified three categories of psychrotolerance: (i) 14 isolates that were not psychrotolerant in BHI or SMB, (ii) 6 isolates that were psychrotolerant in BHI but not in SMB, and (iii) 2 isolates that were psychrotolerant in BHI and SMB. One isolate, which was psychrotolerant in BHI broth but was just below the cut-off of >1 log_10_ cfu/mL increase in SMB was not assigned to a cluster. A maximum likelihood phylogeny constructed with core genome single nucleotide polymorphisms classified all psychrotolerant isolates (i.e., psychrotolerant in BHI) into clade VI (representing *B. mycoides/weihenstephanensis*). Analysis of correlations between gene ortholog presence or absence patterns and psychrotolerance identified 206 orthologous gene clusters that were significantly overrepresented among psychrotolerant strains, including two clusters of cold shock proteins, which were identified in 8/9 and 7/9 psychrotolerant isolates. Gene ontology analyses revealed 36 gene ontology terms that were overrepresented in psychrotolerant isolates, including putrescine catabolic processes and putrescine transmembrane transporter activity. Lastly, Hidden Markov Model searches identified three protein family motifs, including cold shock domain proteins and fatty acid hydroxylases that were significantly associated with psychrotolerance in BHI broth. Analyses of CspA sequences revealed a positive association between psychrotolerant strains and a previously identified “psychrotolerant” CspA sequence. Overall, our data highlight genetic and phenotypic differences in psychrotolerance among *B. cereus* group dairy-associated isolates and show that psychrotolerance is dependent on the growth medium. We also identified a number of gene targets that could be used for specific detection or control of psychrotolerant *B. cereus* group isolates.

## Introduction

Species within the *Bacillus cereus* group are associated with a variety of ecological niches, and are highly diverse in terms of their genetic and phenotypic traits (Guinebretière et al., 2008; Ceuppens et al., 2013). While some species are recognized as human pathogens (e.g., *B. anthracis, B. cereus*) (Didelot et al., 2009; Bottone, 2010), others (e.g., *B. thuringiensis*) are used as insecticides (Höfte and Whiteley, 1989) or as probiotics (e.g., *B. toyonensis*) (Jiménez et al., 2013; Kantas et al., 2015). Some species, in particular *B. weihenstephanensis*, are associated with fluid milk spoilage (Bartoszewicz et al., 2008; Ivy et al., 2012; Hwang and Park, 2015; Saleh-Lakha et al., 2017).

Currently, there are 18 validated species in the *B. cereus* group, with nine being commonly isolated from food (Liu et al., 2017a). Furthermore, WGS data have revealed that multiple named species do not show sufficient genomic differences to actually be considered individual species. For example, species *cereus* and *thuringiensis* should be considered the same genomospecies (i.e., *B. cereus/thuringiensis*), as should be *B. mycoides* and *B. weihenstephanensis* (i.e., *B. mycoides/weihenstephanensis*) (Kovac et al., 2016, 2017; Liu et al., 2017b). Species classification of *B. cereus* group isolates often involves characterization using diagnostic species-specific phenotypic characteristics. For example, isolates with rhizoid morphology are classified as either *B. mycoides* or *B. pseudomycoides* (Nakamura and Jackson, 1995; Nakamura, 1998; Kovac et al., 2016; Miller et al., 2018). While *B. cereus* group species and strains are generally mesophilic (Koehler, 2009; Ceuppens et al., 2013; Jiménez et al., 2013), growth temperature ranges differ between species, which has also been used for species classification, with *B. weihenstephanensis* originally defined by its ability to grow at 7°C but not 43°C (Lechner et al., 1998). While *B. weihenstephanensis* was the first species in the *B. cereus* group that was observed to grow at refrigeration temperatures (Lechner et al., 1998), other *B. cereus* group species have since been reported to grow at or below 7°C (Guinebretière et al., 2008; Soufiane and Côté, 2013; Miller et al., 2016; Liu et al., 2017a). For example, *B. wiedmannii* isolates have been shown to grow at 5–6°C on BHI agar (Miller et al., 2016, 2018). In contrast to *B. weihenstephanensis, B. wiedmannii* isolates are also cytotoxic when cultured at 37°C (Miller et al., 2016, 2018), suggesting this species is a novel pathogen in the *B. cereus* group. Among the *B. cereus* group species, *B. weihenstephanensis* represents the species that is most frequently associated with fluid milk spoilage (Páčová et al., 2003; Bartoszewicz et al., 2008). *B. weihenstephanensis*, as well as potentially other psychrotolerant *Bacillus* spp., thus represent a particular concern for refrigerated dairy products, such as fluid milk (Huck et al., 2008; Ivy et al., 2012; Gopal et al., 2015).

Attempts to identify signature DNA sequences associated with psychrotolerance have previously identified several housekeeping genes (e.g., *gmk, glpF*, and *tpi*) (Francis et al., 1998), as well as genes encoding the conserved cold shock protein CspA that have distinct sequence types for psychrotolerant and mesophilic strains (Lechner et al., 1998). More specifically, comparisons of amino acid sequences of CspA suggested that psychrotolerant strains encode a CspA sequence beginning with “MTV” while mesophilic strains encode for an alanine at the 2^nd^ amino acid (i.e., “MAV”) (Lechner et al., 1998). Other genetic signatures of psychrotolerance include DEAD box RNA helicases, chaperone DnaJ, and the low temperature requirement protein A (LtrA), which were found to be associated with psychrotolerance of *Paenibacillus* spp. isolates in SMB (growth at 6°C for 21 days) (Switt et al., 2014). While associations between psychrotolerant signatures or motifs have been established, it is important to note that phenotypic analyses characterizing the role that these genetic differences may play (such as a conformational change resulting in enhanced function at low temperatures) have not been performed. Fiedoruk et al. (2017) proposed that these motifs could be a reflection of phylogeny, as isolates from phylogenetically related clades have been shown to encode ‘psychrotolerant signatures’ (Fiedoruk et al., 2017). Furthermore, a number of specific adaptations have also been associated with psychrotolerance in bacteria. For example, alterations in RNA metabolism and activity have been linked with psychrotolerance of *Bacillus* spp., presumably as secondary structures that form at low temperatures, which interfere with transcription and translation, ultimately reducing or inhibiting the production of cellular proteins at low temperatures (Barria et al., 2013). More specifically, expression of nucleic acid chaperones, such as cold shock proteins (Csp) has been suggested to aid in enhancing transcription at low temperatures (Gualerzi et al., 2003). The fatty acid composition of the cell membrane has also been shown to be associated with thermotolerance among different species within the *B. cereus* group, with the psychrotolerant groups II (*B. wiedmannii*) and VI (*B. mycoides/weihenstephanensis*) expressing higher ratios of C16:1 fatty acids, compared to the mesophilic clades, when cultured at 30°C (Diomandé et al., 2015).

Based on the importance of psychrotolerant *B. cereus* group species as fluid milk spoilage organisms, and possible concerns about psychrotolerant *B. cereus* group species that have the potential to cause foodborne illness, we performed genetic and phenotypic characterizations on a diverse set of *B. cereus* group isolates grown under conditions that simulate refrigerated fluid milk stored over a 21-day shelf-life. The objective of this study was to assess the psychrotolerance of *B. cereus* group isolates utilizing (i) phenotypic analyses to cluster isolates based on their ability to grow at 6°C in BHI and SMB, and (ii) genetic analyses to associate genes and genetic markers with psychrotolerance. Importantly, we show that psychrotolerance is dependent on the growth medium (i.e., BHI or SMB), and that this growth phenotype is restricted to isolates in clade VI (*B. mycoides/weihenstephanensis*). We also identified a number of genetic markers that are significantly associated with psychrotolerance.

## Materials and Methods

### Isolate Selection

Isolate selection was based on an initial “psychrotolerance” screen of isolates representing all 42 *rpoB* allelic types (AT) (Ivy et al., 2012) found among 503 dairy-associated *B. cereus* group isolates in the Food Safety Lab collection^1^. Clade classification was based on *rpoB* sequence data, which were shown (Kovac et al., 2016) previously to provide for classification consistent with phylogenetic clades proposed by Guinebretière et al. (2008). The initial screen was performed by spread plating 100 μL of overnight cultures grown for 16–20 h at 32°C on BHI agar in two biological replicates. The presence of colonies after a 21-day incubation at 6°C in both biological replicates was considered indicative of psychrotolerance. The screen identified 14 isolates (each representing a different *rpoB* AT) that were psychrotolerant when grown on BHI agar at 6°C for 21 days. All of these isolates were classified into clades II (*B. wiedmannii*) and VI (*B. mycoides/weihenstephanensis*). To allow for comparative studies, we also included nine isolates representing *B. cereus* group subtypes and clades that are not typically considered psychrotolerant. These additional isolates included (i) one isolate that grouped into clade II (*B. wiedmannii*), but was not psychrotolerant when grown at 6°C on BHI agar, and (ii) representative isolates of *B. cereus* group clades I (one isolate; *B. pseudomycoides*), III (three isolates; *B. cereus/anthracis*), and IV [four isolates, including the *B. cereus s.s.* type strain, ATCC 14579^T^ (FSL M8-0473); *B. cereus/thuringiensis*]. No isolates from clades V or VII (representing *B. toyonensis* and *B. cytotoxicus*, respectively) were included in the study, as clade V isolates were rare in our collection of 503 *B. cereus* group dairy isolates, and clade VII isolates were not represented at all in this collection; both of these species have not been previously characterized as being psychrotolerant (Guinebretière et al., 2013; Jiménez et al., 2013).

### Quantification of Growth at 6°C in SMB and BHI Broth

The 23 selected *B. cereus* group isolates were tested for their ability to grow at 6°C in the nutrient-rich BHI broth, and SMB, which we deemed represents conditions similar to fluid milk. Isolates were streaked in triplicate onto BHI agar from frozen glycerol stocks, followed by incubation for 18–24 h at 32°C. One colony of each replicate was inoculated into 5 mL of BHI broth and incubated for 24 h at 32°C (without shaking). These cultures were used to inoculate tubes with 5 mL of pre-chilled BHI or SMB, to achieve an initial concentration of approximately 2.0–3.0 log_10_ cfu/mL (median: 2.7 log_10_ cfu/mL). Tubes were incubated for 21 days (without shaking) at 6°C. On days 0, 14, and 21, a 50 μL aliquot of a serially diluted culture was spiral plated (Autoplate 5000; Advanced Instruments, Inc., Norwood, MA, United States) onto Standard Plate Count (SPC) agar. After spiral plating, SPC agar plates were incubated at 32°C for 24 ± 2 h, colonies were enumerated using a Q-Count Colony Counter (Advanced Instruments, Inc.). Bacterial enumeration data were used to calculate bacterial growth at 6°C after 14 and 21 days, defined as cfu/mL at days 14 and 21, which were then normalized to cfu/mL at day 0. All growth data represent the average of three biological replicates. For data that were below the detection limit of 1 log_10_ cfu/mL, calculations were performed using the detection limit (i.e., 1 log_10_), and the final value was reported as “<” (for absolute numbers) or “>” (for expressing the log reduction over the 2-week incubation) the log_10_ cfu/mL value calculated. Growth patterns were categorized as “psychrotolerant” if an isolate had at least a 1 log_10_ increase in cfu/mL, or “not psychrotolerant” if an isolate either had a decrease in log_10_ cfu/mL, or an increase of < 1 log_10_ cfu/mL, after a 21-day incubation at 6°C.

### Quality Threshold Clustering (QTC)

For quality threshold clustering (QTC) analyses, bacterial counts for days 14 and 21 were normalized to day 0 (as described above) and then averaged for each isolate. The qtclust function of the flexclust package (Leisch, 2006) in R statistical software (R Core Team, 2016) was used to cluster data using a radius of 2.2 log_10_ cfu/mL for 14 and 21 day counts in both BHI and SMB; the radius was chosen to minimize the number of isolates that were not assigned to a cluster and to maximize the number of clusters.

### DNA Extraction and Preparation for Whole Genome Sequencing

Among the 23 isolates included in this study (Table 1), whole genome sequence (WGS) data were already available for eight isolates (Kovac et al., 2016; Miller et al., 2016). For the remaining 15 isolates, WGS was performed as part of the study reported here. For these isolates, DNA was extracted from cultures grown in BHI broth using the QIAamp DNA Mini kit (Qiagen, Valencia, CA, United States) according to a modified protocol that included a 45 min lysis step with 180 μL of 20 mg/mL lysozyme in a 37°C water bath. DNA was eluted in 50 μL of 10 mM Tris-HCl (pH 8.0). The concentration of double-stranded DNA was adjusted to 1 ng/μL, and DNA was then submitted to the Cornell University Institute of Biotechnology Genomics Facility (Ithaca, NY, United States) for Nextera XT DNA library preparation and sequencing. Samples were sequenced in two different Illumina HiSeq runs with 2 bp × 100 bp paired-end reads, having 83 and 113x coverage, respectively.

**Table 1:** Twenty-three *B. cereus* group isolates selected for cold growth quantification and WGS analyses.

Isolate ID	Isolation source	Species identification^a^	WGS accession^b,c^	Clade^d^	*rpoB* AT^e^
FSL H8-0534	Soil, dairy farm	*B. pseudomycoides*	MUAQ00000000	I	148
FSL W8-0169	Raw milk	*B. wiedmannii*	LOBC00000000^c^	II	61
FSL K6-0069	Raw milk	*B. wiedmannii*	LOBB00000000^c^	II	194
FSL M8-0091	Raw milk	*B. wiedmannii*	MUAM00000000	II	410
FSL J3-0113	Pasteurized milk	*B. wiedmannii*	LXFN00000000^b^	II	417
FSL M8-0117	Raw milk	*B. cereus*	LONG00000000^c^	III	308
FSL W8-0483	Raw milk	*B. cereus*	LOMU00000000^c^	III	120
FSL W8-0050	Condensed product in evaporator	*B. cereus*	LOMR00000000^c^	III	125
FSL M8-0473	Farmhouse, 1916	*B. cereus* ATCC 14579^T^	MUAP00000000	IV	158
FSL R5-0811	Pasteurized milk	*B. cereus*	MUAO00000000	IV	158
FSL W8-0268	Evaporator, liquid	*B. cereus*	LOMS00000000^c^	IV	92
FSL K6-1030	Condensed milk	*B. cereus*	MUAU00000000	IV	556
FSL M7-0669	Raw milk	*B. weihenstephanensis*	MUAK00000000	VI	3
FSL H7-0683	Pasteurized milk	*B. mycoides*	MUAR00000000	VI	75
FSL H7-0926	Pasteurized milk	*B. weihenstephanensis*	LOBD00000000^c^	VI	90
FSL M7-1219	Raw milk	*B. weihenstephanensis*	MUAL00000000	VI	97
FSL H8-0485	Soil, dairy farm	*B. weihenstephanensis*	MUAJ00000000	VI	132
FSL H8-0492	Raw milk	*B. weihenstephanensis*	MUAS00000000	VI	134
FSL R5-0708	Pasteurized milk	*B. weihenstephanensis*	MUAN00000000	VI	257
FSL M7-0109	Raw milk	*B. weihenstephanensis*	MUAH00000000	VI	273
FSL J3-0123	Pasteurized milk	*B. weihenstephanensis*	MUAG00000000	VI	513
FSL E2-0214	Pasteurized milk	*B. weihenstephanensis*	MUAT00000000	VI	531
FSL W7-1108	Raw milk	*B. mycoides*	MUAI00000000	VI	342


*^*a*^*B. mycoides* was assigned to isolates in phylogenetic clade VI with rhizoid morphology. ^*b*^Sequenced by Kovac et al. (2016). ^*c*^Sequenced by Miller et al. (2016). ^*d*^Phylogenetic clades were assigned according to WGS clustering (Guinebretière et al., 2008; Kovac et al., 2016). ^*e*^*rpoB* AT represents a subtype based on sequence data for a 632-nt. Fragment of the *rpoB* CDS; isolates that differ by at least 1-nt are assigned different *rpoB* ATs.*

### Read Processing, Quality Control, Genome Assembly, and Annotation

Low quality bases, reads, and Nextera XT adapters were trimmed using the default settings of Trimmomatic v0.33 (Bolger et al., 2014). We assessed short read quality using FastQC (v0.11.2) (Babraham Bioinformatics). Genomes were assembled *de novo* using SPAdes v3.6.2 and a variety of k-mer sizes (21, 33, 55, 77, 99) (Bankevich et al., 2012). QUAST was used to assess the quality of the assembled draft genomes (Gurevich et al., 2013). Average coverage was determined by mapping the reads against draft genomes using BBMap v35.49 and computing the average depth using SAMtools (Li et al., 2009). Sequence reads and assembled draft genomes were submitted to NCBI SRA and WGS databases, respectively (Table 1). Submitted genomes were annotated using the NCBI prokaryotic genome annotation pipeline (Tatusova et al., 2016).

### Single Nucleotide Polymorphism (SNP) Detection and Phylogeny Construction

Single nucleotide polymorphisms (SNPs) were called using kSNP3 (Gardner et al., 2015). The k-mer size of 31 was selected using Kchooser. A maximum likelihood (ML) tree was constructed with RAxML v.8.0 (Stamatakis, 2014) using the core SNPs detected by kSNP3. The ML tree was constructed using a general time-reversible (GTR) model with gamma-distributed sites (GAMMA) and 1000 bootstrap repetitions. Phylogenetic clades were assigned according to WGS clustering (Guinebretière et al., 2008; Kovac et al., 2016). The phylogenetic tree was edited using FigTree v.1.4.2.

### OrthoMCL and Gene Ontology (GO) Term Annotation

All 23 *B. cereus* group isolate genomes were analyzed using OrthoMCL (Li et al., 2003) with an inflation value of 2.5 to find groups of orthologous genes found across multiple isolates (i.e., ortholog clusters). A representative protein sequence for each of the 9,885 identified clusters was used for GO annotation using Blast2GO (Conesa and Götz, 2008) searches against SWISS-PROT and RefSeq databases. All protein sequences were searched against the SWISS-PROT database (Bairoch and Apweiler, 2000) and only protein sequences with no GO terms mapped to the SWISS-PROT database were searched against the RefSeq database (O’Leary et al., 2015). The outputs from SWISS-PROT and RefSeq searches were combined and the assigned GO terms were linked to their respective ortholog clusters and to each member of the ortholog cluster. OrthoMCL clusters that were overrepresented among psychrotolerant isolates were assigned gene names by running BLAST using the protein sequences against RefSeq and SWISS-PROT.

### Gene Presence/Absence Analysis and Gene Enrichment

Using the ortholog clusters from the OrthoMCL output, the counts of genomes where each gene was present or absent were computed for isolates classified as “psychrotolerant” or “not psychrotolerant” in BHI broth. Presence/absence data for each OrthoMCL cluster (gene) were used to generate 2x2 tables, which were analyzed using two-sided Fisher’s exact tests. Odds ratios were computed, and *p*-values were adjusted using the false discovery rate (FDR) method.

To identify GO terms that were over- or under-represented among genomes of isolates that were classified as “psychrotolerant” or “not psychrotolerant” in BHI, the number of genes classified as a given GO term were summed for each genome, and then for all genomes classified as showing “psychrotolerant” or “not psychrotolerant.” This approach was used to generate 2x2 tables for each GO term, which were used to run two-sided Fisher’s exact tests and compute odds ratios as described above. FDR-adjusted *p*-values < 0.05 were considered statistically significant.

### Cold Shock Protein Sequence Analysis and Classification

Amino acid sequences of putative cold shock proteins (Csp) identified by SWISS-PROT and Refseq were compared against previously published sequences for five unique Csp proteins from *B. cereus* and *B. subtilis*, namely CspA, CspB, CspC, CspD, CspE (Mayr et al., 1996; Francis et al., 1998; Schindler et al., 1999) and an uncharacterized Csp protein described in Mayr et al. (1996). In addition, the distribution of mesophilic and psychrotolerant variants of CspA were characterized by classifying strains based on their having the mesophilic CspA sequence (beginning with “MAV”) or the psychrotolerant sequence (beginning with “MTV”) (Mayr et al., 1996; Francis et al., 1998). In addition, BLAST searches were performed for all Csp proteins using reference sequences for named Csp genes and variants described previously (Mayr et al., 1996; Francis et al., 1998; Fiedoruk et al., 2017) as well as two additional reference sequences representing (i) a novel Csp divergently related to CspC and (ii) a novel Csp annotated by the NCBI pipeline in one of the isolates sequenced here (FSL W8-0050). An e-value of 10^-30^, with ≤5 amino acid differences between the reference and query sequence match over the full length of the subject sequence, were used as criteria for determining the presence of Csp genes and alleles.

Fisher’s exact tests were used to test for an association between psychrotolerance and the presence of the psychrotolerant CspA sequence. *p*-Values < 0.05 were considered statistically significant.

### Identification of Proteins Related to Psychrotolerance

Twelve HMM protein domains previously shown to be associated with psychrotolerance were obtained from Pfam 26.0 protein families’ database (see Supplementary Table S1; Finn et al., 2015). HMMER v.3.2 (Eddy, 2011), with default parameters was used to search these HMM models against the genomes for the 23 isolates characterized here. Linear regression was performed using R statistical software (2016) to determine whether protein family matches per genome are associated with psychrotolerance after a 21-day incubation at 6°C (calculated as log_10_ cfu/mL at day 21 – log_10_ cfu/mL at day 0).

## Results

### QTC Analyses Identify Three General Psychrotolerance Patterns for *B. cereus* Group Isolates From Clades Commonly Isolated From Dairy Foods and Environments

**FIGURE 1:**
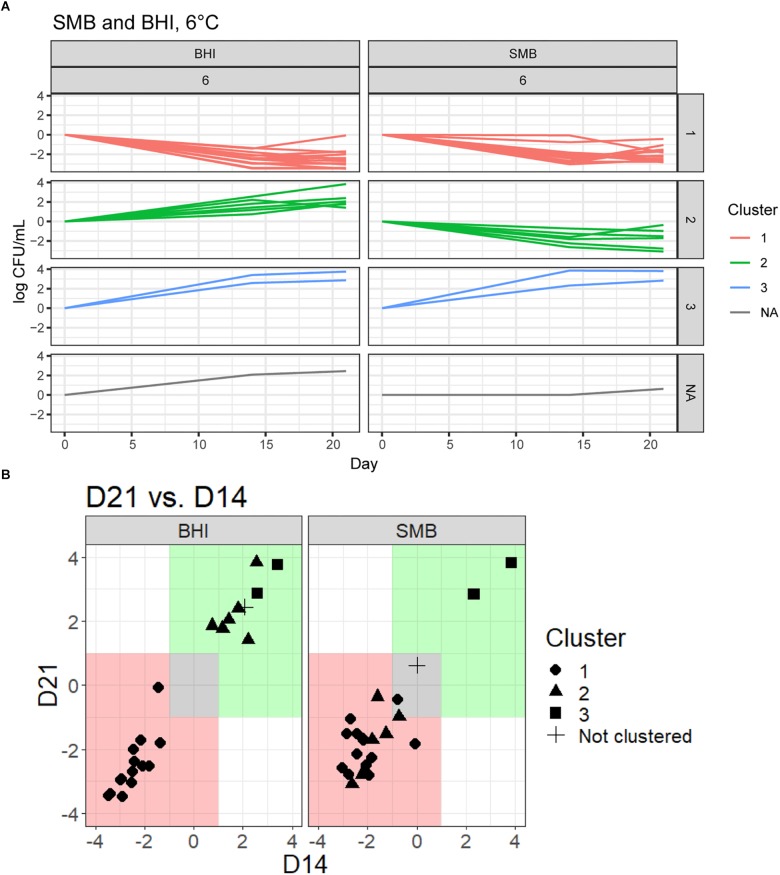
Growth patterns in SMB and BHI broth at 6°C for 23 *Bacillus cereus* group isolates. **(A)** Isolates were grouped according to growth in both SMB and BHI broth using quality threshold clustering (QTC). Growth data of three replicates were averaged for each isolate and growth patterns for each isolate were clustered according to their growth under these conditions, clusters are color coded according to their growth patterns. Cluster 1, marked in red, is comprised of 14 isolates that generally showed vegetative cell reduction in both BHI and SMB at 6°C. Isolates (*n* = 6) in cluster 2, marked in green, generally showed >1 log_10_ increase in cfu/mL at either day 14 or day 21 in BHI broth, and <1 log_10_ cfu/mL increase in SMB. Cluster 3, marked in blue, is comprised of two isolates that showed >1 log_10_ increase in cfu/mL in both BHI and SMB. There was also one isolate, in gray, that did not group using QTC. **(B)** Growth data (relative to day 0) at each time point were averaged for each of the 23 isolates (three replicates) and plotted for day 14 on the x-axis and for day 21 on the y-axis. Growth patterns for both BHI and SMB are displayed. Different shapes (e.g., circle, triangle) on the graph indicate each QTC group. The area in green represents >1 log_10_ growth, while the area in red represents >1 log_10_ reduction. The area shaded in gray represents <1 log_10_ difference (increase or decrease) from starting inoculum concentration.

**FIGURE 2:**
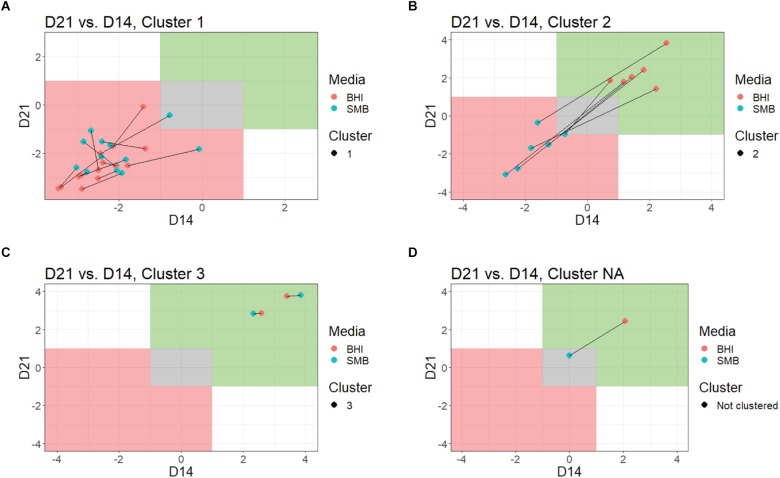
Comparison of growth in BHI broth and SMB, by QTC cluster. Growth data at each time point were plotted for each QTC, individually. Lines connect data from the same isolates, red dots indicate the day 14 vs. day 21 bacterial counts in BHI, blue dots indicating the day 14 vs. day 21 bacterial counts in SMB. The green shaded area is indicative of growth (>1 log_10_ increase), while gray represents no growth and red indicates reduction (>1 log_10_ decrease). Data for QTC clusters 1 **(A)**, 2 **(B)**, 3 **(C)**, and isolate FSL M7-0109, which was not assigned to a QTC cluster **(D)** are shown.

To objectively categorize *B. cereus* group isolates based on their ability to grow under conditions that simulate refrigerated fluid milk (SMB) and a nutrient rich media (BHI broth) we used QTC analyses to group isolates with similar growth patterns, independent of their phylogenetic relationship. Isolates generally showed consistent growth or reduction at both days 14 and 21 (Figure 1 and Table 2). QTC analyses identified three clusters (Figure 1A, 2A–D) of growth patterns after 21 days incubation at 6°C: cluster 1, containing isolates that were not psychrotolerant in BHI or SMB (i.e., <1 log_10_ increase in cfu/mL in both media); cluster 2, containing isolates that were psychrotolerant in BHI but not SMB, and cluster 3 containing isolates that were psychrotolerant in both SMB and BHI broth. One isolate classified into clade VI (*B. mycoides/weihenstephanensis*; FSL M7-0109) was not assigned to a cluster; this isolate was psychrotolerant in BHI but was just below the >1 log_10_ cfu/mL cut-off for psychrotolerance in SMB (Figure 2D).

**Table 2:** Log difference in bacterial count for 14 and 21 days after incubation at 6°C in BHI and SMB.

Isolate	Clade	BHI			SMB			
				
		Day 14 – 0 (log_10_ cfu/mL)	Day 21 – 0 (log_10_ cfu/mL)	Psychro-tolerant^a^	Day 14 – 0 (log_10_ cfu/mL)	Day 21 – 0 (log_10_ cfu/mL)	Psychro-tolerant^a^	QTC cluster^b^
FSL W8-0169	II	> -1.5	> -1.9	No	-0.1	> -1.2	No	1
FSL H7-0683	VI	> -1.1	-0.07	No	> -1.5	> -1.4	No	1
FSL H7-0926	VI	> -1.5	> -1.4	No	-0.8	-0.4	No	1
FSL M8-0091	II	> -2.0	> -2.0	No	> -1.8	> -1.8	No	1
FSL J3-0113	II	> -2.2	> -2.5	No	> -1.6	> -1.8	No	1
FSL K6-1030	IV	> -1.4	> -1.4	No	> -1.4	> -1.5	No	1
FSL W8-0483	III	> -1.9	> -2.1	No	> -1.4	> -1.7	No	1
FSL M8-0117	III	> -1.2	> -1.0	No	> -1.2	> -1.0	No	1
FSL W8-0050	III	> -1.9	> -1.9	No	> -1.5	> -1.6	No	1
FSL K6-0069	II	> -1.8	> -2.0	No	> -1.7	-1.1	No	1
FSL R5-0811	IV	> -2.4	> -2.4	No	> -1.8	> -1.8	No	1
FSL M8-0473	IV	> -1.8	> -1.9	No	> -1.9	-1.6	No	1
FSL W8-0268	IV	> -2.5	> -2.5	No	> -2.0	> -1.9	No	1
FSL H8-0534	I	> -0.7	> -0.8	No	> -1.4	> -1.2	No	1
FSL H8-0485	VI	1.2	1.8	Yes	> -2.0	> -2.1	No	2
FSL H8-0492	VI	1.4	2.1	Yes	-1.3	> -1.2	No	2
FSL R5-0708	VI	2.6	3.8	Yes	> -0.9	> -0.0	No	2
FSL W7-1108	VI	2.2	1.4	Yes	> -1.1	> -1.0	No	2
FSL J3-0123	VI	0.7	1.9	Yes	-0.7	-1.0	No	2
FSL E2-0214	VI	1.8	2.4	Yes	> -1.6	> -1.8	No	2
FSL M7-0669	VI	3.4	3.8	Yes	3.9	3.8	Yes	3
FSL M7-1219	VI	2.6	2.9	Yes	2.3	2.8	Yes	3
FSL M7-0109	VI	2.1	2.4	Yes	0.0	0.6	No	NA


*^*a*^Psychrotolerant: isolates that showed at least a 1 log_*10*_ increase after 14- or 21-days incubation at 6°C in SMB or BHI. ^*b*^NA: this isolate was not assigned to a QTC cluster*.

Quality threshold clustering cluster 1 isolates (*n* = 14) had an average of >1.7 log_10_ reduction in BHI, and >1.4 log_10_ reduction in SMB, after a 21-day incubation at 6°C (Figure 1, 2A and Table 2). All isolates from clades I (*B. pseudomycoides*), II (*B. wiedmannii*), III (*B. anthracis/cereus*), IV (*B. cereus/thuringiensis*), and 2/11 isolates from clade VI (*B. mycoides/weihenstephanensis*) were assigned to QTC cluster 1.

Six isolates [all from clade VI (*B. mycoides/ weihenstephanensis*)] were categorized into QTC cluster 2 (Figure 1, 2B and Table 2), which represented isolates that were psychrotolerant in BHI but not in SMB after 21 days at 6°C. All isolates in this cluster showed growth of at least 1 log_10_ cfu/mL in BHI at both 14 and 21 days (average growth of 1.7 and 2.2 log_10_ cfu/mL after 14 and 21 days, respectively). In SMB, these isolates had an average of >1.3 and >1.2 log_10_ cfu/mL reductions after 14 and 21 days, respectively.

Quality threshold clustering cluster 3 (Figure 1, 2C and Table 2) contains two isolates that were psychrotolerant in both BHI and SMB. Both isolates belonged to clade VI (*B. mycoides/weihenstephanensis*), and grew in both BHI (average growth of 3.0 and 3.4 log_10_ after 14 and 21 days, respectively) and SMB (3.1 and 3.3 log_10_ increase after 14 and 21 days) incubated at 6°C.

### Psychrotolerance in BHI Broth Is Unique to Isolates in Clade VI

**FIGURE 3:**
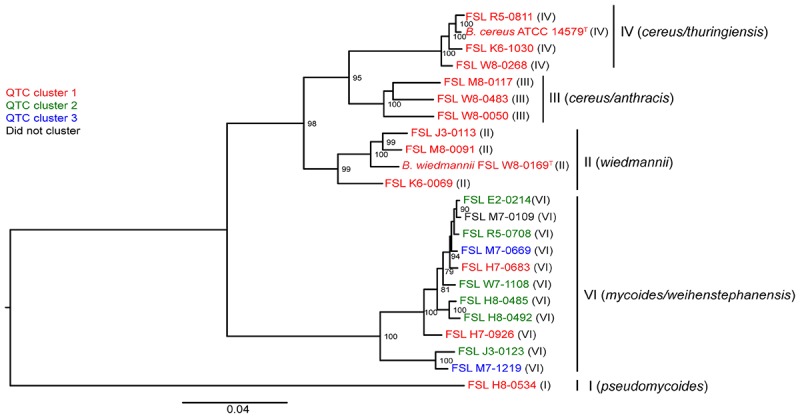
Phylogenetic tree constructed from the core SNPs identified in the genomes of 23 *B. cereus* isolates. The maximum likelihood tree was constructed using a general time-reversible (GTR) model with gamma-distributed sites and 1,000 bootstrap repetitions. Roman numerals in parentheses represent the phylogenetic clade of the isolate, as defined previously (Kovac et al., 2016). QTC clusters representing isolates with similar growth patterns in BHI and SMB at 6°C (see Figure 1A) are mapped onto the phylogenetic tree. Isolates shown in red represent QTC cluster 1 isolates (non-psychrotolerant in BHI broth or SMB). Isolates shown in green represent QTC cluster 2 isolates (psychrotolerant in BHI broth but not SMB), and isolates shown in blue represent clade 3 isolates (psychrotolerant in both BHI broth and SMB). One isolate (FSL M7-0109) did not cluster with other isolates, and is therefore shown in black font. Numbers at branch points represent bootstrap values; only bootstrap values >70 are shown.

To assess whether there was a relationship between phylogenetic clustering and psychrotolerance, we first mapped the QTC clusters onto a phylogenetic tree based on core genome SNPs of the 23 isolates included in this study. All psychrotolerant isolates clustered in phylogenetic clade VI (*B. mycoides/weihenstephanensis*); this clade also contained two isolates that were not psychrotolerant in either SMB or BHI (Figure 3). All tested isolates from the other clades (i.e., clades I, II, III, and IV) consistently showed reduction in cell counts in both SMB (mean decrease of >1.5 log_10_ after 14 and 21 days) and BHI at 6°C (mean decrease of >1.8 and >1.9 log_10_ after 14 and 21 days, respectively).

### Targeted Searches for Protein Families and Gene Variants Previously Associated With Psychrotolerance Identified Genetic Markers for Growth at 6°C Among *B. cereus* Group Isolates

To test for specific genetic markers associated with psychrotolerance, we first performed HMM searches for 12 protein families that had previously been identified as being associated with psychrotolerance and cold adaptation. Members of 11 of these 12 protein families were identified in at least one of the *B. cereus* group genomes tested (see Supplementary Table S2 for details). For eight protein families, genes encoding for these proteins were identified in all 23 genomes, although the number of distinct hits in a given genome sometimes differed considerably (Supplementary Table S2). For example, either six or seven distinct hits for the cold shock domain (listed as “CSD” in Supplementary Table S2) were identified among each of the 23 different genomes. Isolates in QTC cluster 2, which were psychrotolerant in BHI but not in SMB, averaged 6.8 distinct hits whereas all other isolates averaged 6.3 distinct hits. The three HMM families for which we found distinct hits in less than 23 genomes included LtrA, Caps synth-CapC, and FA desaturase 2. LtrA was identified in only two genomes [FSL M8-0473 (ATCC 14579^T^) and FSL R5-0811], which are both clade IV (*B. cereus/thuringiensis*) isolates. Caps synth-CapC and FA desaturase 2 were found at least once in 22/23 and 20/23 genomes, respectively (see Supplementary Table S2 for details).

For three HMM, we found a significant positive association between the number of hits and psychrotolerance in BHI; these families include cold shock domain proteins (*p* = 0.013), FA hydroxylase (*p* = 0.011), and YdjO (*p* = 0.002). Psychrotolerant isolates averaged 6.8, 2.1, and 2.7 hits for cold shock domain, FA hydroxylase, and YdjO members, while non-psychrotolerant isolates averaged 6.2, 1.5, and 1.9 distinct hits, respectively. Furthermore, isolates from QTC cluster 2 (psychrotolerant in BHI but not in SMB) averaged more hits of these protein families than isolates in QTC clusters 1 or 3. Surprisingly, the HMM representing the DEAD domain showed a significant negative association (*p* = 0.025) with psychrotolerance. Isolates categorized as “not psychrotolerant” averaged more hits for the DEAD domain (37.5 hits), than psychrotolerant isolates (35.9 hits).

We also performed BLAST searches for individual named cold shock proteins (CspA, CspB, CspC, CspD, and CspE) and one protein identified through OrthoMCL analyses (defined as “CspM” here) and one protein that was identified as a Csp when the isolate (FSL W8-0050) was annotated using the NCBI annotation pipeline (Supplementary Table S7). Analyses revealed a significant association (*p* = 0.003; Fisher’s exact test) between psychrotolerant isolates and the presence of the gene encoding the previously defined psychrotolerant CspA signature (Francis et al., 1998). The psychrotolerant CspA sequence was found in 12 isolates, including all 11 clade VI isolates (with 9/11 isolates classified as psychrotolerant) and the 1 clade I isolate (*B. pseudomycoides*) tested (which was classified as not psychrotolerant). The remaining 11 *B. cereus* genomes encoded the mesophilic CspA sequence. CspC and CspE (as described in Fiedoruk et al., 2017) were detected in all isolates, regardless of phylogenetic clade. CspB was detected in all clade I and VI isolates, and half of the clade II isolates. CspD1 and CspD2 (Fiedoruk et al., 2017) were detected in all isolates, with clade II and VI isolates encoding CspD1_IIa-c and D2_IV-M, and isolates in clades III and IV encoding CpsD1_IV-I/M and CpsD2_IV-M; the clade I (*B. pseudomycoides*) isolate encoded the CspD1_IV-I/M and the CspD2_IV-M variants. CpsM was detected in eight isolates (all clade VI) and one isolate encoded CspN (Supplementary Table S7).

### Genes Encoding for Invasion Proteins, Cold Shock Proteins, and Protein Families Involved in Putrescine Metabolism Are Overrepresented Among *B. cereus* Group Isolates That Are Psychrotolerant in BHI Broth

To search for novel genes associated with the observed psychrotolerance we also used OrthoMCL to compare gene presence/absence between psychrotolerant isolates (able to grow at 6°C in BHI broth) and non-psychrotolerant isolates. Overall, OrthoMCL identified 9,885 different orthologous clusters (i.e., clusters of genes assumed to encode for proteins that share the same characteristics) among the 23 genomes analyzed. FDR-corrected Fisher’s exact tests were used to identify genes that were overrepresented among the nine isolates that were psychrotolerant in BHI broth; we did not test for genes overrepresented among isolates that were psychrotolerant in SMB, as only two isolates fit this category. These analyses revealed 206 orthologous gene clusters significantly overrepresented in isolates that were psychrotolerant in BHI broth, and 182 orthologous gene clusters significantly overrepresented in isolates that were not psychrotolerant in BHI broth (Supplementary Tables S3, S4). Importantly, we did not identify any orthologous gene clusters that were unique to all 9 isolates that were psychrotolerant in BHI broth, but were absent from all 14 isolates that were not psychrotolerant in BHI broth. However, we identified 87 clusters (including some discussed in more detail below) that were found among all nine isolates that were psychrotolerant in BHI broth, and only found in 2/14 isolates that were not; all of these gene clusters were specific for clade VI (*B. mycoides/weihenstephanensis*) and were only found among the 11 clade VI strains characterized here.

Two orthologous clusters representing cold shock proteins (cluster 4979 and cluster 5279) were overrepresented in psychrotolerant isolates (Supplementary Table S3). These clusters were identified as genes encoding YdjO (98% coverage to *B. subtilis* YdjO) and CspC (100% coverage to *B. subtilis* CspC), respectively. Further comparison of the amino acid sequence from *B. cereus* CspC against the newly identified sequence showed that the new sequence does not correspond to *B. cereus* CspC and was therefore re-named “CspM.” *ydjO* (cluster 4979) was identified in 8/9 isolates that were psychrotolerant in BHI broth (all except for FSL W7-1108, *B. mycoides/weihenstephanensis*), while *cspM* (cluster 5279) was identified in 7/9 psychrotolerant isolates (all except for FSL H8-0492 and FSL M7-0669, both *B. mycoides/weihenstephanensis*). Interestingly, *ydjO* was found in two isolates that were not psychrotolerant in BHI broth (FSL H7-0926 and FSL H7-0683) and *cspM* was identified in one isolate (FSL H7-0926) that was not psychrotolerant in BHI broth. Both of these genes were only identified in isolates from phylogenetic clade VI (*B. mycoides/weihenstephanensis*), regardless of whether or not the isolate was psychrotolerant; specifically, *ydjO* and *cspM* were found in 10/11 and 8/11 clade VI isolates, respectively.

Other orthologous clusters overrepresented in the nine psychrotolerant isolates include: (i) two genes encoding invasion proteins (clusters 4691 and 4799), (ii) two genes annotated as encoding homologs of the damage inducible protein DinB (clusters 4457 and 4953), (iii) two genes encoding glyoxalases (clusters 4700 and 4937), and (iv) two genes encoding GNAT family acetyltransferases (clusters 4699 and 4966) (Supplementary Table S3). Both orthologous clusters encoding for invasion proteins were found in all nine psychrotolerant isolates and in the two non-psychrotolerant isolates (FSL H7-0926 and FSL H7-0683) that are also in clade VI (*B. mycoides/weihenstephanensis*); no specific pre-existing gene names could be assigned to these two clusters. Orthologous clusters 4457 and 4953, which were annotated as the damage-inducible protein DinB, showed 98 and 99% coverage to *dinB* in several species in the *B. cereus* group, respectively. Cluster 4457 was identified in all psychrotolerant strains, as well as in three isolates that were not-psychrotolerant in BHI broth; these three isolates (all clade II, *B. wiedmannii*) however showed growth at 6°C on BHI agar (Miller et al., 2018). Cluster 4457 was identified in all 11 clade VI isolates, and in isolate FSL J3-0113 (clade II, *B. wiedmannii*). Cluster 4953 was identified in 8/9 isolates that were psychrotolerant in BHI broth (all except for FSL H8-0482) and in 2/14 isolates that were not psychrotolerant in BHI broth (FSL H7-0926 and FSL H7-0683). Overall, cluster 4953 was identified in 10/11 isolates classified into phylogenetic clade VI (*B. mycoides/weihenstephanensis*). Among the two orthologous clusters annotated as glyoxalases, no specific gene name could be assigned to cluster 4937, but cluster 4700 was identified as *mhqA* (98% coverage in *B. subtilis* with equal coverage matches to paralogs *mhqE* and *mqoO*), which encodes a putative ring-cleaving dioxygenase. Cluster 4937 was found in 8/9 isolates that were psychrotolerant in BHI broth (all expect for FSL W7-1108), while cluster 4700 was found in all isolates that were psychrotolerant in BHI broth. Cluster 4937 and cluster 4700 were found in 10/11 and 11/11 clade VI (*B. mycoides/weihenstephanensis*) isolates, respectively. Among the two clusters identified as GNAT family acetyltransferases, cluster 4699 showed 97% coverage to YoaA of *B. subtilis*, while cluster 4966 could not be assigned a specific gene name (no coverage match > 95%). *yoaA* was present in all nine psychrotolerant isolates and in two non-psychrotolerant isolates, while cluster 4966 was identified in 8/9 psychrotolerant isolates and in two non-psychrotolerant isolates. *yoaA* was found in all 11 clade VI isolates while cluster 4966 was identified in 10/11 clade VI (*B. mycoides/weihenstephanensis*) isolates (excluding FSL M7-1219); neither of these clusters were identified in isolates outside of clade VI.

GO term analyses revealed 36 terms that were significantly overrepresented in isolates psychrotolerant in BHI broth, and 12 terms that were significantly overrepresented in isolates that were not psychrotolerant in BHI broth (see Supplementary Tables S5, S6 for details). GO terms found to be overrepresented in the genomes of *B. cereus* group isolates classified as psychrotolerant in BHI broth included the terms “putrescine catabolic process” (GO:0009447) and “putrescine transmembrane transporter activity” (GO:0015489) (see Supplementary Table S5 for a complete list). GO terms found to be overrepresented in the genomes of *B. cereus* group isolates that were not psychrotolerant in BHI broth include “alkanesulfonate transporter activity” (GO:0042959), “alkanesulfonate monooxygenase activity” (GO:0008726), and “alkanesulfonate catabolic process” (GO:0046306) (see Supplementary Table S6 for a complete list).

## Discussion

The *B. cereus* group includes multiple species that are frequently isolated from dairy foods, including foodborne pathogens (e.g., *B. cereus* strains expressing emetic or diarrheal toxins) and spoilage organisms, which are of particular importance for refrigerated dairy foods such as fluid milk (e.g., *B. weihenstephanensis*). A number of recent studies (Kovac et al., 2016; Warda et al., 2016; Zhang et al., 2017; Miller et al., 2018) have combined phenotypic and genomic-based approaches to allow for improved characterization and identification of *B. cereus* group clonal groups, strains, and isolates that have the ability to cause human disease. However, these studies did not examine the genetic basis for psychrotolerance among *B. cereus* group isolates. An improved understanding of psychrotolerance and the underlying genetic markers that are associated would allow for improved (i) risk assessments, (ii) detection and characterization methods that can rapidly detect and identify *B. cereus* group strains that can grow at refrigeration temperatures, and (iii) development of control strategies, specifically for refrigerated dairy products (e.g., fluid milk, certain types of high pH cheeses) that may permit *B. cereus* group isolates’ growth. Our results provide important data characterizing psychrotolerance among *B. cereus* group isolates obtained from dairy-associated sources. Our data specifically indicate that among *B. cereus* species isolates commonly isolated from dairy foods and dairy environments, (i) psychrotolerance in liquid media seems to be limited to strains in *B. cereus* group clade VI (*B. mycoides/weihenstephanensis*) and that (ii) a number of genes are linked to psychrotolerance of *B. cereus* group isolates.

### Psychrotolerance in SMB or Rich Media Is Limited to Some, but Not All, Isolates in Phylogenetic Clade VI (*B. mycoides/weihenstephanensis*)

While our initial screen identified 14 *B. cereus* group isolates that showed growth on BHI agar at 6°C [11 and 3 isolates from clades VI (*B. mycoides/weihenstephanensis*) and II (*B. wiedmannii*), respectively], only nine of these isolates showed growth in BHI broth at 6°C, and just two isolates grew in SMB incubated at 6°C. While 3/4 isolates in phylogenetic clade II (*B. wiedmannii*), were able to grow on BHI agar incubated at 5–6°C (Miller et al., 2016, 2018), none of these isolates were classified as psychrotolerant in BHI broth or SMB in the results presented here. Interestingly, 4/4 *B. wiedmannii* isolates included in this study encoded the mesophilic variant of the CspA sequence, whereas the psychrotolerant CspA sequence was only found in the *B. mycoides/weihenstephanensis* isolates, and the one *B. pseudomycoides* isolate. *B. wiedmannii* was first described in 2016 as a psychrotolerant member of the *B. cereus* group (Miller et al., 2016) where 5/11 isolates showed growth after 21 days of incubation at 5°C on BHI agar and all strains showed growth at 10°C. This suggests that the ability to grow at 6°C may differ between planktonic versus surface growth for this species and suggests a need for further characterization of *B. wiedmannii* for its ability to grow in different dairy products (e.g., fluid milk, fresh cheeses) under refrigeration conditions, as recent studies (Kovac et al., 2016; Miller et al., 2016, 2018) have shown that *B. wiedmannii* isolates carry virulence toxin genes associated with diarrheal disease (*hblACD* and *nheABC*) and show cytotoxicity in a HeLa cell culture model (Miller et al., 2016, 2018). Additionally, *B. wiedmannii* isolates have been shown to produce both Hbl and Nhe toxins (Kovac et al., 2016; Miller et al., 2016, 2018). Future studies are needed to combine growth and virulence data to better assess the foodborne illness risk associated with *B. wiedmannii* contamination of different dairy products.

While nine isolates, which were all classified into *B. cereus* group clade VI (*B. mycoides/weihenstephanensis*), showed growth at 6°C in BHI broth, only two of these isolates were also psychrotolerant in SMB. Future experiments may be needed to characterize and compare the ability of psychrotolerant Bacillales to grow in SMB and fluid milk in order to better assess food safety and spoilage risks associated with the presence of different Bacillales (e.g., *Paenibacillus, B. wiedmannii, B. weihenstephanensis*) in fluid milk. This is important as autoclaving of SMB, as performed here, may result in the production of Maillard reaction products, which have been linked to antimicrobial activity (Ledl and Schleicher, 1990; Hauser et al., 2014). Therefore, SMB may prevent growth of *B. cereus* group isolates under certain conditions, even though growth may occur in fluid milk. Clade VI (*B. mycoides/weihenstephanensis*), included all nine isolates that were psychrotolerant in BHI broth. While a number of *B. cereus* group species have been reported to include isolates that grow at low temperatures (Stenfors and Granum, 2001; Soufiane and Côté, 2013; Miller et al., 2016), our data are consistent with previous reports that *B. weihenstephanensis* isolates typically are able to grow at refrigeration temperatures (Lechner et al., 1998) and observations that isolates classified into this species have repeatedly been linked to spoilage issues in fluid milk (Páčová et al., 2003; Ivy et al., 2012; Trmčić et al., 2015). Likewise, our data demonstrate the ability of *B. mycoides* isolates to grow at 6°C. *B. mycoides* was previously identified as capable of growing at 7°C (Guinebretière et al., 2008), but is not classically defined as being psychrotolerant. Meanwhile, *B. weihenstephanensis*, which was discovered more than a century after *B. mycoides* was described in 1886 (Lewis, 1932), has been described as psychrotolerant (Lechner et al., 1998; Stenfors and Granum, 2001). Importantly, WGS shows that *B. weihenstephanensis* and *B. mycoides* should be considered the same genomospecies [despite *B. mycoides* having a rhizhoid morphology (Kovac et al., 2016; Miller et al., 2018)], which would consolidate these Bacillales that are typically psychrotolerant into a single species. It’s noteworthy though, that among the 11 clade VI (*B. mycoides/weihenstephanensis*) isolates tested here, only two did not grow in either BHI or SMB. Previous studies have also found that not all *B. weihenstephanensis* show growth at low temperatures. For example, a 2008 study by Guinebretière et al. (2008) reported 7/143 *B. weihenstephanensis* isolates were unable to grow at 7°C on J-agar.

All isolates outside of clade VI (*B. mycoides/weihenstephanensis*) showed reductions in vegetative cell numbers in both BHI and SMB after incubation for 14 to 21 days at 6°C, as did some clade VI isolates, particularly when incubated in SMB. While these findings may suggest die-off, reduction of vegetative cell numbers, as determined by plating on SPC agar, could also be caused by *B. cereus* group cells entering spore form during exposure to cold stress. Although sporulation due to cold stress has not been studied in *Bacillus* spp., a characterization study of thermophilic *Clostridium thermocellum* JW20 found that vegetative cells entered spore form when the temperature fell below 45°C for several hours (Freier et al., 1988). In contrast, a more recent study of *C. thermocellum* found that another strain (ATTC 27405) does not enter spore form at temperatures below 48°C (Mearls et al., 2012), suggesting that sporulation from sub-optimum temperature stress may be a strain-dependent phenotype. Future experiments will thus be needed to assess sporulation of different Bacillales isolates under cold stress exposure, as well as the environmental signals required for these cells to re-enter the vegetative state.

### Overrepresentation of Selected Genes Linked to Psychrotolerance in Phylogenetic Clade VI (*B. mycoides/weihenstephanensis*) Isolates Identifies Potential Targets for Detection of Isolates More Likely to Grow at Low Temperatures

Overall, 206 genes identified using OrthoMCL and three domains identified by HMM had a significant positive association with *B. cereus* group isolates that were psychrotolerant in BHI broth. Cold shock proteins have been studied in the *B. cereus* group (Brillard et al., 2010), and a signature sequence of one particular cold shock gene, *cspA* (encoding CspA) has been used previously to identify *B. weihenstephanensis* (Francis et al., 1998; Lechner et al., 1998; Stenfors and Granum, 2001). In our study we found a significant association between psychrotolerant isolates and the psychrotolerant CspA signature sequence consistent with a previous study (Lechner et al., 1998). It is important however to note that all isolates that were psychrotolerant in BHI broth in this study were from phylogenetic clade VI (*B. mycoides/weihenstephanensis*). The CspA signature was also found in a clade I *B. pseudomycoides* isolate (FSL H8-0534), despite this strain not being psychrotolerant in BHI broth or SMB. It is interesting to note that *B. pseudomycoides* isolates have not been previously observed to grow at 6°C. Of similar interest, none of the clade II isolates (*B. wiedmannii*) harbored the psychrotolerant CspA sequence, despite their being psychrotolerant when grown at 6°C on BHI agar. A recent study by Fiedoruk et al. (2017) suggested that the psychrotolerant signature sequences could be a reflection of the phylogenetic ancestry of the strain, rather than an adaptation enabling psychrotolerance. However, further phylogenetic analyses would be required in order to determine the evolutionary history of the *B. cereus* group species and confirm this hypothesis. Interestingly, analysis of additional cold shock proteins (as detailed by Fiedoruk et al., 2017) among the 23 isolates listed here revealed that all isolates encode CspC and CspE, and therefore detection of these genes does not represent a useful screening method for detection of psychrotolerant strains of *B. cereus*. Furthermore, we identified two novel cold shock proteins, CspM and CspN (see Supplementary Table S7). CspM was detected in 8/11 clade VI isolates, and CspN was detected in just one clade III strain. Further mutagenesis studies of the psychrotolerant and mesophilic motifs in CspA as well as different cold shock protein genes is needed to determine whether certain motifs or genes enable psychrotolerance or whether their presence solely represent a marker of phylogenetic relatedness and facilitates phenotypes other than the ability to grow at low temperatures.

Aside from the psychrotolerant CspA variant, we also identified a number of (i) protein family motifs and (ii) genes associated with psychrotolerant *B. cereus* group isolates. HMM analyses revealed three protein families for which the number of hits was positively correlated with isolates that were psychrotolerant in BHI broth, including cold shock domain proteins, fatty acid hydroxylases, and the YdjO protein domain. Cold shock domain proteins have previously been associated with isolates that can grow at low temperatures, especially *B. weihenstephanensis* (Francis et al., 1998; Lechner et al., 1998). Fatty acid hydroxylases, which have altered expression at low temperatures to allow for adjustments in membrane fluidity, have also been previously associated with psychrotolerance (Mansilla et al., 2004; Chan and Wiedmann, 2008; Diomandé et al., 2015). Finally, the YdjO domain is not only found in the cold shock-induced YdjO protein, which is functionally uncharacterized (Kaan et al., 2002), but is also found in other proteins and may be linked to the ability to grow at lower temperatures. More “hits” of these protein families (cold shock domain, fatty acid hydroxylases, and YdjO) were found in isolates that were psychrotolerant in BHI broth. Surprisingly, DEAD box RNA helicases, which have been previously reported to be associated with psychrotolerance among Bacillales genera (Chan and Wiedmann, 2008; Barria et al., 2013; Switt et al., 2014), had more “hits” among isolates included in this study that were not psychrotolerant in BHI. Genes identified as overrepresented in psychrotolerant *B. cereus* group isolates included *ydjO* and *cspM*, both encoding cold shock proteins. Further functional studies confirming the roles of *ydjO* and *cspM* in psychrotolerance, will be beneficial to assess whether these genes represent targets appropriate for rapid identification of psychrotolerant *B. cereus* strains.

Overall, we identified a number of genetic targets associated with *B. cereus* group isolates that are psychrotolerant in BHI broth. Future work characterizing the expression and role of these genes in psychrotolerance will be beneficial for predictive assays aimed at detecting psychrotolerant *B. cereus* group isolates. As psychrotolerant *B. cereus* group isolates represent important spoilage bacteria in dairy foods, the genetic targets identified here could be used to develop improved DNA-based assays for rapid and specific identification of psychrotolerant *B. cereus* group isolates throughout the dairy supply chain. Furthermore, our data also demonstrate the importance of assessing psychrotolerance using multiple methods, which, in the context of food isolates, may include media representative of different foods as well as different growth conditions (e.g., liquid culture vs. agar plates).

## Conclusion

Isolates in the *B. cereus* group demonstrate differing capacities for psychrotolerance, which is dependent on phylogeny. While previous studies have suggested that psychrotolerant isolates exist across a variety of *B. cereus* group species (Stenfors and Granum, 2001; Soufiane and Côté, 2013; Miller et al., 2016), phenotypic characterization of *B. cereus* group isolates representing the predominant sequence types in dairy foods and environments showed that only isolates from phylogenetic clade VI (*B. mycoides/weihenstephanensis*) showed growth at 6°C in either SMB or BHI broth. Genetic analyses revealed a number of genes and GO terms significantly associated with isolates that were psychrotolerant in BHI broth, and confirmed that the psychrotolerant CspA amino acid motif is associated with psychrotolerant *B. cereus* group isolates. While the presence of the psychrotolerant CspA amino acid sequence was positively associated with *B. cereus* group isolates psychrotolerant in BHI broth, it is important to consider that in this study, only clade VI isolates showed growth at 6°C in either BHI broth or SMB. All clade VI isolates had the psychrotolerant CspA amino acid sequence, regardless of the isolate’s ability to grow at 6°C. The clade I *B. pseudomycoides* isolate, FSL H8-0534, which did not grow at 6°C in either BHI or SMB, also had the psychrotolerant CspA amino acid sequence. Meanwhile, *B. wiedmannii*, a previously described psychrotolerant species with pathogenic potential did not have the psychrotolerant CspA sequence. In addition to future research on the functional importance of different genetic markers associated with psychrotolerance in *B. cereus* groups species, further work on growth at refrigeration temperatures in different matrices and under different environmental conditions will be important, particularly since our data suggest that *B. wiedmannii* can grow at 6°C on solid media, but not liquid substrates tested.

## Data Availability

The datasets generated for this study can be found in NCBI, https://www.ncbi.nlm.nih.gov/bioproject?LinkName=nuccore_bioproject&from_uid=1151525537.

## Author Contributions

SB, NM, JK, and MW designed the study. SB and DD conducted growth experiments. SB, RO, DK, JK, and RC performed bioinformatical and statistical analyses. SB, RO, RC, and MW wrote the manuscript. All authors assisted with the revision of this manuscript.

## Conflict of Interest Statement

The authors declare that the research was conducted in the absence of any commercial or financial relationships that could be construed as a potential conflict of interest.

## Footnotes

1 http://www.foodmicrobetracker.com
