# Corrigendum: Genes Associated With Psychrotolerant *Bacillus cereus* Group Isolates

**DOI:** 10.3389/fmicb.2019.01323

**Published:** 2019-06-12

**Authors:** Sarah M. Beno, Renato H. Orsi, Rachel A. Cheng, David J. Kent, Jasna Kovac, Diana R. Duncan, Nicole H. Martin, Martin Wiedmann

**Affiliations:** ^1^Department of Food Science, Cornell University, Ithaca, NY, United States; ^2^Department of Food Science, Penn State University, University Park, PA, United States; ^3^Department of Food Science, Wageningen University, Wageningen, Netherlands

**Keywords:** *Bacillus cereus*, whole genome sequencing, psychrotolerant, spoilage, skim milk broth

In the original article, there were two minor mistakes in Figure 3 as published. The isolate FSL H8-0534 was mistakenly labeled as a clade VI isolate. We have updated the figure to demonstrate that this isolate is a clade I isolate. Additionally, isolate FSL M7-1219 had an additional “0” in the number sequence. The corrected Figure 3 appears below.

**Figure 3:**
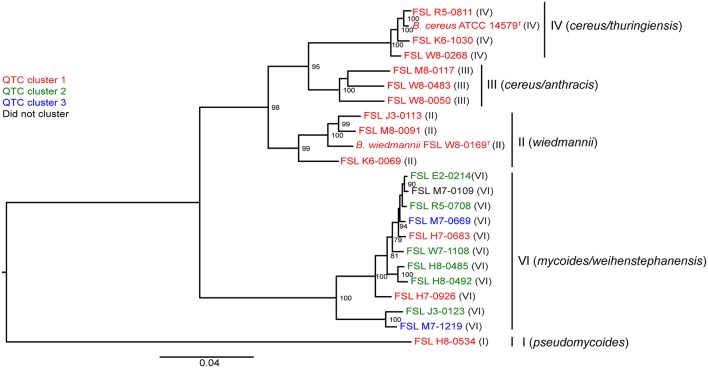
Phylogenetic tree constructed from the core SNPs identified in the genomes of 23 *B. cereus* isolates. The maximum likelihood tree was constructed using a general time-reversible (GTR) model with gamma-distributed sites and 1,000 bootstrap repetitions. Roman numerals in parentheses represent the phylogenetic clade of the isolate, as defined previously (Kovac et al., 2016). QTC clusters representing isolates with similar growth patterns in BHI and SMB at 6°C (see Figure 3A) are mapped onto the phylogenetic tree. Isolates shown in red represent QTC cluster 1 isolates (non-psychrotolerant in BHI broth or SMB). Isolates shown in green represent QTC cluster 2 isolates (psychrotolerant in BHI broth but not SMB), and isolates shown in blue represent clade 3 isolates (psychrotolerant in both BHI broth and SMB). One isolate (FSL M7-0109) did not cluster with other isolates, and is therefore shown in black font. Numbers at branch points represent bootstrap values; only bootstrap values >70 are shown.

The authors apologize for these errors and state that this does not change the scientific conclusions of the article in any way. The original article has been updated.
