# Supplementary material for: Genes Associated With Psychrotolerant Bacillus cereus Group Isolates
Source: Front Microbiol. 2019 Mar 29;10:662. doi: 10.3389/fmicb.2019.00662 (PMC6449464; doi:10.3389/fmicb.2019.00662)
Supplement: Supplementary file 1 [file Table_1.DOCX]

**Supplemental Table 1**: Twelve proteins or protein domains previous associated with growth at low temperatures used for Hidden Markov Model (HMM) analyses.

| Query | Accession | Description | Length (aa) |
| --- | --- | --- | --- |
| Caps_synth_CapC | PF1402.5 | Capsule biosynthesis CapC | 119 |
| CSD | PF00313.21 | Cold shock DNA-binding domain | 66 |
| DEADboxA | PF12343.7 | Cold shock protein DEAD box A | 69 |
| DEAD | PF00270.28 | DEAD/DEAH box helicase | 176 |
| DnaJ | PF00226.30 | DnaJ domain | 63 |
| FA_desaturase_2 | PF03405.13 | Fatty acid desaturase | 326 |
| FA_desaturase | PF00487.23 | Fatty acid desaturase | 254 |
| FA_hydroxylase | PF04116.12 | Fatty acid hydroxylase superfamily | 133 |
| LtrA | PF06772.10 | Bacterial low temperature requirement A protein (LtrA) | 353 |
| Peptidase_S11 | PF00768.19 | D-alanyl-D-alanine carboxypeptidase | 241 |
| RecA | PF00154.20 | RecA bacterial DNA recombination protein | 263 |
| YdjO | PF14169.5 | Cold-inducible protein YdjO | 59 |
