# Supplementary material for: Genes Associated With Psychrotolerant Bacillus cereus Group Isolates
Source: Front Microbiol. 2019 Mar 29;10:662. doi: 10.3389/fmicb.2019.00662 (PMC6449464; doi:10.3389/fmicb.2019.00662)
Supplement: Supplementary file 2 [file Table_2.DOCX]

Supplemental Table 2: Hidden Markov Model (HMM) results for 23 *B. cereus* group isolates

| Query | Accession | Strain | Count Match Per Genome^a^ | Count Genomes^b^ |
| --- | --- | --- | --- | --- |
| Caps_synth_CapC | PF14102.5 | FSL_E2-0214 | 2 | - |
| Caps_synth_CapC | PF14102.5 | FSL_H7-0683 | 2 | - |
| Caps_synth_CapC | PF14102.5 | FSL_H7-0926 | 2 | - |
| Caps_synth_CapC | PF14102.5 | FSL_H8-0485 | 2 | - |
| Caps_synth_CapC | PF14102.5 | FSL_H8-0492 | 2 | - |
| Caps_synth_CapC | PF14102.5 | FSL_H8-0534 | 1 | - |
| Caps_synth_CapC | PF14102.5 | FSL_J3-0113 | 2 | - |
| Caps_synth_CapC | PF14102.5 | FSL_K6-0069 | 3 | - |
| Caps_synth_CapC | PF14102.5 | FSL_K6-1030 | 1 | - |
| Caps_synth_CapC | PF14102.5 | FSL_M7-0109 | 2 | - |
| Caps_synth_CapC | PF14102.5 | FSL_M7-0669 | 2 | - |
| Caps_synth_CapC | PF14102.5 | FSL_M7-1219 | 1 | - |
| Caps_synth_CapC | PF14102.5 | FSL_M8-0091 | 1 | - |
| Caps_synth_CapC | PF14102.5 | FSL_M8-0117 | 1 | - |
| Caps_synth_CapC | PF14102.5 | FSL_M8-0473 | 2 | - |
| Caps_synth_CapC | PF14102.5 | FSL_R5-0708 | 3 | - |
| Caps_synth_CapC | PF14102.5 | FSL_R5-0811 | 3 | - |
| Caps_synth_CapC | PF14102.5 | FSL_W7-1108 | 1 | - |
| Caps_synth_CapC | PF14102.5 | FSL_W8-0050 | 2 | - |
| Caps_synth_CapC | PF14102.5 | FSL_W8-0169 | 2 | - |
| Caps_synth_CapC | PF14102.5 | FSL_W8-0268 | 3 | - |
| Caps_synth_CapC | PF14102.5 | FSL_W8-0483 | 2 | 22 |
| CSD | PF00313.21 | FSL_E2-0214 | 7 | - |
| CSD | PF00313.21 | FSL_H7-0683 | 6 | - |
| CSD | PF00313.21 | FSL_H7-0926 | 7 | - |
| CSD | PF00313.21 | FSL_H8-0485 | 7 | - |
| CSD | PF00313.21 | FSL_H8-0492 | 6 | - |
| CSD | PF00313.21 | FSL_H8-0534 | 6 | - |
| CSD | PF00313.21 | FSL_J3-0113 | 6 | - |
| CSD | PF00313.21 | FSL_J3-0123 | 7 | - |
| CSD | PF00313.21 | FSL_K6-0069 | 6 | - |
| CSD | PF00313.21 | FSL_K6-1030 | 6 | - |
| CSD | PF00313.21 | FSL_M7-0109 | 7 | - |
| CSD | PF00313.21 | FSL_M7-0669 | 6 | - |
| CSD | PF00313.21 | FSL_M7-1219 | 7 | - |
| CSD | PF00313.21 | FSL_M8-0091 | 6 | - |
| CSD | PF00313.21 | FSL_M8-0117 | 6 | - |
| CSD | PF00313.21 | FSL_M8-0473 | 6 | - |
| CSD | PF00313.21 | FSL_R5-0708 | 7 | - |
| CSD | PF00313.21 | FSL_R5-0811 | 6 | - |
| CSD | PF00313.21 | FSL_W7-1108 | 7 | - |
| CSD | PF00313.21 | FSL_W8-0050 | 7 | - |
| CSD | PF00313.21 | FSL_W8-0169 | 7 | - |
| CSD | PF00313.21 | FSL_W8-0268 | 6 | - |
| CSD | PF00313.21 | FSL_W8-0483 | 6 | 23 |
| DEAD | PF00270.28 | FSL_E2-0214 | 36 | - |
| DEAD | PF00270.28 | FSL_H7-0683 | 33 | - |
| DEAD | PF00270.28 | FSL_H7-0926 | 35 | - |
| DEAD | PF00270.28 | FSL_H8-0485 | 37 | - |
| DEAD | PF00270.28 | FSL_H8-0492 | 37 | - |
| DEAD | PF00270.28 | FSL_H8-0534 | 36 | - |
| DEAD | PF00270.28 | FSL_J3-0113 | 39 | - |
| DEAD | PF00270.28 | FSL_J3-0123 | 35 | - |
| DEAD | PF00270.28 | FSL_K6-0069 | 41 | - |
| DEAD | PF00270.28 | FSL_K6-1030 | 39 | - |
| DEAD | PF00270.28 | FSL_M7-0109 | 39 | - |
| DEAD | PF00270.28 | FSL_M7-0669 | 34 | - |
| DEAD | PF00270.28 | FSL_M7-1219 | 35 | - |
| DEAD | PF00270.28 | FSL_M8-0091 | 36 | - |
| DEAD | PF00270.28 | FSL_M8-0117 | 39 | - |
| DEAD | PF00270.28 | FSL_M8-0473 | 35 | - |
| DEAD | PF00270.28 | FSL_R5-0708 | 34 | - |
| DEAD | PF00270.28 | FSL_R5-0811 | 38 | - |
| DEAD | PF00270.28 | FSL_W7-1108 | 36 | - |
| DEAD | PF00270.28 | FSL_W8-0050 | 40 | - |
| DEAD | PF00270.28 | FSL_W8-0169 | 37 | - |
| DEAD | PF00270.28 | FSL_W8-0268 | 36 | - |
| DEAD | PF00270.28 | FSL_W8-0483 | 41 | 23 |
| DnaJ | PF00226.30 | FSL_E2-0214 | 1 | - |
| DnaJ | PF00226.30 | FSL_H7-0683 | 2 | - |
| DnaJ | PF00226.30 | FSL_H7-0926 | 1 | - |
| DnaJ | PF00226.30 | FSL_H8-0485 | 3 | - |
| DnaJ | PF00226.30 | FSL_H8-0492 | 1 | - |
| DnaJ | PF00226.30 | FSL_H8-0534 | 4 | - |
| DnaJ | PF00226.30 | FSL_J3-0113 | 2 | - |
| DnaJ | PF00226.30 | FSL_J3-0123 | 2 | - |
| DnaJ | PF00226.30 | FSL_K6-0069 | 3 | - |
| DnaJ | PF00226.30 | FSL_K6-1030 | 2 | - |
| DnaJ | PF00226.30 | FSL_M7-0109 | 1 | - |
| DnaJ | PF00226.30 | FSL_M7-0669 | 1 | - |
| DnaJ | PF00226.30 | FSL_M7-1219 | 2 | - |
| DnaJ | PF00226.30 | FSL_M8-0091 | 2 | - |
| DnaJ | PF00226.30 | FSL_M8-0117 | 1 | - |
| DnaJ | PF00226.30 | FSL_M8-0473 | 1 | - |
| DnaJ | PF00226.30 | FSL_R5-0708 | 1 | - |
| DnaJ | PF00226.30 | FSL_R5-0811 | 1 | - |
| DnaJ | PF00226.30 | FSL_W7-1108 | 2 | - |
| DnaJ | PF00226.30 | FSL_W8-0050 | 2 | - |
| DnaJ | PF00226.30 | FSL_W8-0169 | 2 | - |
| DnaJ | PF00226.30 | FSL_W8-0268 | 1 | - |
| DnaJ | PF00226.30 | FSL_W8-0483 | 3 | 23 |
| FA_desaturase | PF00487.23 | FSL_E2-0214 | 7 | - |
| FA_desaturase | PF00487.23 | FSL_H7-0683 | 7 | - |
| FA_desaturase | PF00487.23 | FSL_H7-0926 | 7 | - |
| FA_desaturase | PF00487.23 | FSL_H8-0485 | 10 | - |
| FA_desaturase | PF00487.23 | FSL_H8-0492 | 8 | - |
| FA_desaturase | PF00487.23 | FSL_H8-0534 | 8 | - |
| FA_desaturase | PF00487.23 | FSL_J3-0113 | 8 | - |
| FA_desaturase | PF00487.23 | FSL_J3-0123 | 7 | - |
| FA_desaturase | PF00487.23 | FSL_K6-0069 | 9 | - |
| FA_desaturase | PF00487.23 | FSL_K6-1030 | 9 | -- |
| FA_desaturase | PF00487.23 | FSL_M7-0109 | 8 | - |
| FA_desaturase | PF00487.23 | FSL_M7-0669 | 6 | - |
| FA_desaturase | PF00487.23 | FSL_M7-1219 | 5 | - |
| FA_desaturase | PF00487.23 | FSL_M8-0091 | 9 | - |
| FA_desaturase | PF00487.23 | FSL_M8-0117 | 7 | - |
| FA_desaturase | PF00487.23 | FSL_M8-0473 | 6 | - |
| FA_desaturase | PF00487.23 | FSL_R5-0708 | 6 | - |
| FA_desaturase | PF00487.23 | FSL_R5-0811 | 6 | - |
| FA_desaturase | PF00487.23 | FSL_W7-1108 | 8 | - |
| FA_desaturase | PF00487.23 | FSL_W8-0050 | 9 | - |
| FA_desaturase | PF00487.23 | FSL_W8-0169 | 6 | - |
| FA_desaturase | PF00487.23 | FSL_W8-0268 | 8 | - |
| FA_desaturase | PF00487.23 | FSL_W8-0483 | 6 | 23 |
| FA_desaturase_2 | PF03405.13 | FSL_E2-0214 | 1 | - |
| FA_desaturase_2 | PF03405.13 | FSL_H7-0683 | 1 | - |
| FA_desaturase_2 | PF03405.13 | FSL_H7-0926 | 2 | - |
| FA_desaturase_2 | PF03405.13 | FSL_H8-0485 | 3 | - |
| FA_desaturase_2 | PF03405.13 | FSL_H8-0492 | 1 | - |
| FA_desaturase_2 | PF03405.13 | FSL_H8-0534 | 2 | - |
| FA_desaturase_2 | PF03405.13 | FSL_J3-0113 | 1 | - |
| FA_desaturase_2 | PF03405.13 | FSL_K6-0069 | 2 | - |
| FA_desaturase_2 | PF03405.13 | FSL_K6-1030 | 3 | - |
| FA_desaturase_2 | PF03405.13 | FSL_M7-0109 | 1 | - |
| FA_desaturase_2 | PF03405.13 | FSL_M7-0669 | 2 | - |
| FA_desaturase_2 | PF03405.13 | FSL_M7-1219 | 2 | - |
| FA_desaturase_2 | PF03405.13 | FSL_M8-0091 | 1 | - |
| FA_desaturase_2 | PF03405.13 | FSL_M8-0473 | 2 | - |
| FA_desaturase_2 | PF03405.13 | FSL_R5-0708 | 1 | - |
| FA_desaturase_2 | PF03405.13 | FSL_R5-0811 | 1 | - |
| FA_desaturase_2 | PF03405.13 | FSL_W7-1108 | 4 | - |
| FA_desaturase_2 | PF03405.13 | FSL_W8-0050 | 2 | - |
| FA_desaturase_2 | PF03405.13 | FSL_W8-0169 | 2 | - |
| FA_desaturase_2 | PF03405.13 | FSL_W8-0268 | 1 | 20 |
| FA_hydroxylase | PF04116.12 | FSL_E2-0214 | 2 | - |
| FA_hydroxylase | PF04116.12 | FSL_H7-0683 | 3 | - |
| FA_hydroxylase | PF04116.12 | FSL_H7-0926 | 2 | - |
| FA_hydroxylase | PF04116.12 | FSL_H8-0485 | 2 | - |
| FA_hydroxylase | PF04116.12 | FSL_H8-0492 | 2 | - |
| FA_hydroxylase | PF04116.12 | FSL_H8-0534 | 3 | - |
| FA_hydroxylase | PF04116.12 | FSL_J3-0113 | 1 | - |
| FA_hydroxylase | PF04116.12 | FSL_J3-0123 | 2 | - |
| FA_hydroxylase | PF04116.12 | FSL_K6-0069 | 1 | - |
| FA_hydroxylase | PF04116.12 | FSL_K6-1030 | 1 | - |
| FA_hydroxylase | PF04116.12 | FSL_M7-0109 | 2 | - |
| FA_hydroxylase | PF04116.12 | FSL_M7-0669 | 2 | - |
| FA_hydroxylase | PF04116.12 | FSL_M7-1219 | 1 | - |
| FA_hydroxylase | PF04116.12 | FSL_M8-0091 | 2 | - |
| FA_hydroxylase | PF04116.12 | FSL_M8-0117 | 1 | - |
| FA_hydroxylase | PF04116.12 | FSL_M8-0473 | 1 | - |
| FA_hydroxylase | PF04116.12 | FSL_R5-0708 | 3 | - |
| FA_hydroxylase | PF04116.12 | FSL_R5-0811 | 1 | - |
| FA_hydroxylase | PF04116.12 | FSL_W7-1108 | 3 | - |
| FA_hydroxylase | PF04116.12 | FSL_W8-0050 | 1 | - |
| FA_hydroxylase | PF04116.12 | FSL_W8-0169 | 2 | - |
| FA_hydroxylase | PF04116.12 | FSL_W8-0268 | 1 | - |
| FA_hydroxylase | PF04116.12 | FSL_W8-0483 | 1 | 23 |
| LtrA | PF06772.10 | FSL_M8-0473 | 1 | - |
| LtrA | PF06772.10 | FSL_R5-0811 | 1 | 2 |
| Peptidase_S11 | PF00768.19 | FSL_E2-0214 | 11 | - |
| Peptidase_S11 | PF00768.19 | FSL_H7-0683 | 11 | - |
| Peptidase_S11 | PF00768.19 | FSL_H7-0926 | 13 | - |
| Peptidase_S11 | PF00768.19 | FSL_H8-0485 | 13 | - |
| Peptidase_S11 | PF00768.19 | FSL_H8-0492 | 12 | - |
| Peptidase_S11 | PF00768.19 | FSL_H8-0534 | 13 | - |
| Peptidase_S11 | PF00768.19 | FSL_J3-0113 | 12 | - |
| Peptidase_S11 | PF00768.19 | FSL_J3-0123 | 16 | - |
| Peptidase_S11 | PF00768.19 | FSL_K6-0069 | 14 | - |
| Peptidase_S11 | PF00768.19 | FSL_K6-1030 | 13 | - |
| Peptidase_S11 | PF00768.19 | FSL_M7-0109 | 13 | - |
| Peptidase_S11 | PF00768.19 | FSL_M7-0669 | 11 | - |
| Peptidase_S11 | PF00768.19 | FSL_M7-1219 | 16 | - |
| Peptidase_S11 | PF00768.19 | FSL_M8-0091 | 12 | - |
| Peptidase_S11 | PF00768.19 | FSL_M8-0117 | 11 | - |
| Peptidase_S11 | PF00768.19 | FSL_M8-0473 | 11 | - |
| Peptidase_S11 | PF00768.19 | FSL_R5-0708 | 11 | - |
| Peptidase_S11 | PF00768.19 | FSL_R5-0811 | 12 | - |
| Peptidase_S11 | PF00768.19 | FSL_W7-1108 | 16 | - |
| Peptidase_S11 | PF00768.19 | FSL_W8-0050 | 13 | - |
| Peptidase_S11 | PF00768.19 | FSL_W8-0169 | 13 | - |
| Peptidase_S11 | PF00768.19 | FSL_W8-0268 | 11 | - |
| Peptidase_S11 | PF00768.19 | FSL_W8-0483 | 15 | 23 |
| RecA | PF00154.20 | FSL_E2-0214 | 3 | - |
| RecA | PF00154.20 | FSL_H7-0683 | 3 | - |
| RecA | PF00154.20 | FSL_H7-0926 | 3 | - |
| RecA | PF00154.20 | FSL_H8-0485 | 3 | - |
| RecA | PF00154.20 | FSL_H8-0492 | 3 | - |
| RecA | PF00154.20 | FSL_H8-0534 | 6 | - |
| RecA | PF00154.20 | FSL_J3-0113 | 2 | - |
| RecA | PF00154.20 | FSL_J3-0123 | 3 | - |
| RecA | PF00154.20 | FSL_K6-0069 | 3 | - |
| RecA | PF00154.20 | FSL_K6-1030 | 4 | - |
| RecA | PF00154.20 | FSL_M7-0109 | 3 | - |
| RecA | PF00154.20 | FSL_M7-0669 | 5 | - |
| RecA | PF00154.20 | FSL_M7-1219 | 3 | - |
| RecA | PF00154.20 | FSL_M8-0091 | 4 | - |
| RecA | PF00154.20 | FSL_M8-0117 | 1 | - |
| RecA | PF00154.20 | FSL_M8-0473 | 4 | - |
| RecA | PF00154.20 | FSL_R5-0708 | 3 | - |
| RecA | PF00154.20 | FSL_R5-0811 | 4 | - |
| RecA | PF00154.20 | FSL_W7-1108 | 3 | - |
| RecA | PF00154.20 | FSL_W8-0050 | 3 | - |
| RecA | PF00154.20 | FSL_W8-0169 | 3 | - |
| RecA | PF00154.20 | FSL_W8-0268 | 3 | - |
| RecA | PF00154.20 | FSL_W8-0483 | 2 | 23 |
| YdjO | PF14169.5 | FSL_E2-0214 | 3 | - |
| YdjO | PF14169.5 | FSL_H7-0683 | 3 | - |
| YdjO | PF14169.5 | FSL_H7-0926 | 3 | - |
| YdjO | PF14169.5 | FSL_H8-0485 | 3 | - |
| YdjO | PF14169.5 | FSL_H8-0492 | 3 | - |
| YdjO | PF14169.5 | FSL_H8-0534 | 2 | - |
| YdjO | PF14169.5 | FSL_J3-0113 | 2 | - |
| YdjO | PF14169.5 | FSL_J3-0123 | 2 | - |
| YdjO | PF14169.5 | FSL_K6-0069 | 3 | - |
| YdjO | PF14169.5 | FSL_K6-1030 | 1 | - |
| YdjO | PF14169.5 | FSL_M7-0109 | 3 | - |
| YdjO | PF14169.5 | FSL_M7-0669 | 3 | - |
| YdjO | PF14169.5 | FSL_M7-1219 | 2 | - |
| YdjO | PF14169.5 | FSL_M8-0091 | 2 | - |
| YdjO | PF14169.5 | FSL_M8-0117 | 1 | - |
| YdjO | PF14169.5 | FSL_M8-0473 | 1 | - |
| YdjO | PF14169.5 | FSL_R5-0708 | 3 | - |
| YdjO | PF14169.5 | FSL_R5-0811 | 1 | - |
| YdjO | PF14169.5 | FSL_W7-1108 | 2 | - |
| YdjO | PF14169.5 | FSL_W8-0050 | 1 | - |
| YdjO | PF14169.5 | FSL_W8-0169 | 2 | - |
| YdjO | PF14169.5 | FSL_W8-0268 | 2 | - |
| YdjO | PF14169.5 | FSL_W8-0483 | 1 | 23 |

^a^ “Count Match Per Genome” refers to the number of times a domain is found in a given genome

^b^ “Count Genomes” is the total of genomes in which a given domain query is found
