# Supplementary material for: Genes Associated With Psychrotolerant Bacillus cereus Group Isolates
Source: Front Microbiol. 2019 Mar 29;10:662. doi: 10.3389/fmicb.2019.00662 (PMC6449464; doi:10.3389/fmicb.2019.00662)
Supplement: Supplementary file 3 [file Table_3.DOCX]

**Supplemental Table 3**: OrthoMCL clusters: List of gene clusters significantly associated with psychrotolerant isolates (>1log_10_ increase after 21-day incubation at 6°C in BHI broth)

| Cluster | Number of Genes | Number of Taxa | Presence among Psychrot-olerant Strains | Absence among Psychr-otolerant Strains | Presence among Non-Psychrotolerant Strains | Absence among Non-Psychrotolerant Strains | P-value*^a^* | FDR*^b^* | Odds Ratio*^c^* | Products |
| --- | --- | --- | --- | --- | --- | --- | --- | --- | --- | --- |
| Cluster_5932 | 6 | 6 | 6 | 3 | 0 | 14 | 0.0008 | 0.0267 | Inf | 2'-5' RNA ligase |
| Cluster_5930 | 6 | 6 | 6 | 3 | 0 | 14 | 0.0008 | 0.0267 | Inf | hypothetical protein |
| Cluster_5931 | 6 | 6 | 6 | 3 | 0 | 14 | 0.0008 | 0.0267 | Inf | hypothetical protein |
| Cluster_5933 | 6 | 6 | 6 | 3 | 0 | 14 | 0.0008 | 0.0267 | Inf | saccharopine dehydrogenase |
| Cluster_5934 | 6 | 6 | 6 | 3 | 0 | 14 | 0.0008 | 0.0267 | Inf | transcriptional regulator |
| Cluster_5661 | 7 | 7 | 7 | 2 | 0 | 14 | 0.0001 | 0.0113 | Inf | carboxymuconolactone decarboxylase family |
| Cluster_5663 | 7 | 7 | 7 | 2 | 0 | 14 | 0.0001 | 0.0113 | Inf | hypothetical protein |
| Cluster_5279 | 8 | 8 | 7 | 2 | 1 | 13 | 0.0010 | 0.0316 | 34.39 | cold-shock protein |
| Cluster_5269 | 8 | 8 | 7 | 2 | 1 | 13 | 0.0010 | 0.0316 | 34.39 | hypothetical protein |
| Cluster_5277 | 8 | 8 | 7 | 2 | 1 | 13 | 0.0010 | 0.0316 | 34.39 | hypothetical protein |
| Cluster_5301 | 8 | 8 | 7 | 2 | 1 | 13 | 0.0010 | 0.0316 | 34.39 | hypothetical protein |
| Cluster_5374 | 8 | 8 | 7 | 2 | 1 | 13 | 0.0010 | 0.0316 | 34.39 | hypothetical protein |
| Cluster_5375 | 8 | 8 | 7 | 2 | 1 | 13 | 0.0010 | 0.0316 | 34.39 | hypothetical protein |
| Cluster_5376 | 8 | 8 | 7 | 2 | 1 | 13 | 0.0010 | 0.0316 | 34.39 | hypothetical protein |
| Cluster_5377 | 8 | 8 | 7 | 2 | 1 | 13 | 0.0010 | 0.0316 | 34.39 | hypothetical protein |
| Cluster_5378 | 8 | 8 | 7 | 2 | 1 | 13 | 0.0010 | 0.0316 | 34.39 | hypothetical protein |
| Cluster_5380 | 8 | 8 | 7 | 2 | 1 | 13 | 0.0010 | 0.0316 | 34.39 | hypothetical protein |
| Cluster_5381 | 8 | 8 | 7 | 2 | 1 | 13 | 0.0010 | 0.0316 | 34.39 | hypothetical protein |
| Cluster_5384 | 8 | 8 | 7 | 2 | 1 | 13 | 0.0010 | 0.0316 | 34.39 | hypothetical protein |
| Cluster_5388 | 8 | 8 | 7 | 2 | 1 | 13 | 0.0010 | 0.0316 | 34.39 | hypothetical protein |
| Cluster_5391 | 8 | 8 | 7 | 2 | 1 | 13 | 0.0010 | 0.0316 | 34.39 | hypothetical protein |
| Cluster_5389 | 8 | 8 | 7 | 2 | 1 | 13 | 0.0010 | 0.0316 | 34.39 | NUDIX hydrolase |
| Cluster_5390 | 8 | 8 | 7 | 2 | 1 | 13 | 0.0010 | 0.0316 | 34.39 | polysaccharide deacetylase |
| Cluster_5382 | 8 | 8 | 7 | 2 | 1 | 13 | 0.0010 | 0.0316 | 34.39 | RNA polymerase subunit sigma-24 |
| Cluster_5379 | 8 | 8 | 7 | 2 | 1 | 13 | 0.0010 | 0.0316 | 34.39 | transcriptional regulator |
| Cluster_4940 | 10 | 9 | 8 | 1 | 1 | 13 | 0.0002 | 0.0113 | 65.56 | alpha/beta hydrolase |
| Cluster_5086 | 9 | 9 | 8 | 1 | 1 | 13 | 0.0002 | 0.0113 | 65.56 | cyclic pyranopterin monophosphate synthase |
| Cluster_5189 | 9 | 9 | 8 | 1 | 1 | 13 | 0.0002 | 0.0113 | 65.56 | hypothetical protein |
| Cluster_5190 | 9 | 9 | 8 | 1 | 1 | 13 | 0.0002 | 0.0113 | 65.56 | hypothetical protein |
| Cluster_5194 | 9 | 9 | 8 | 1 | 1 | 13 | 0.0002 | 0.0113 | 65.56 | hypothetical protein |
| Cluster_5195 | 9 | 9 | 8 | 1 | 1 | 13 | 0.0002 | 0.0113 | 65.56 | hypothetical protein |
| Cluster_5193 | 9 | 9 | 8 | 1 | 1 | 13 | 0.0002 | 0.0113 | 65.56 | phosphatase |
| Cluster_5187 | 9 | 9 | 8 | 1 | 1 | 13 | 0.0002 | 0.0113 | 65.56 | recombinase RecQ |
| Cluster_4939 | 10 | 10 | 8 | 1 | 2 | 12 | 0.0007 | 0.0238 | 36.21 | acetyltransferase |
| Cluster_4945 | 10 | 10 | 8 | 1 | 2 | 12 | 0.0007 | 0.0238 | 36.21 | acetyltransferase |
| Cluster_4972 | 10 | 10 | 8 | 1 | 2 | 12 | 0.0007 | 0.0238 | 36.21 | acetyltransferase |
| Cluster_4979 | 10 | 10 | 8 | 1 | 2 | 12 | 0.0007 | 0.0238 | 36.21 | cold-shock protein |
| Cluster_4978 | 10 | 10 | 8 | 1 | 2 | 12 | 0.0007 | 0.0238 | 36.21 | copper oxidase |
| Cluster_4967 | 10 | 10 | 8 | 1 | 2 | 12 | 0.0007 | 0.0238 | 36.21 | cupin |
| Cluster_4953 | 10 | 10 | 8 | 1 | 2 | 12 | 0.0007 | 0.0238 | 36.21 | damage-inducible protein DinB |
| Cluster_4968 | 10 | 10 | 8 | 1 | 2 | 12 | 0.0007 | 0.0238 | 36.21 | dihydrolipoamide dehydrogenase |
| Cluster_4925 | 10 | 10 | 8 | 1 | 2 | 12 | 0.0007 | 0.0238 | 36.21 | DNA mismatch repair protein MutT |
| Cluster_4922 | 10 | 10 | 8 | 1 | 2 | 12 | 0.0007 | 0.0238 | 36.21 | flavoprotein |
| Cluster_4937 | 10 | 10 | 8 | 1 | 2 | 12 | 0.0007 | 0.0238 | 36.21 | glyoxalase |
| Cluster_4966 | 10 | 10 | 8 | 1 | 2 | 12 | 0.0007 | 0.0238 | 36.21 | GNAT family acetyltransferases |
| Cluster_4934 | 10 | 10 | 8 | 1 | 2 | 12 | 0.0007 | 0.0238 | 36.21 | group-specific protein |
| Cluster_4955 | 10 | 10 | 8 | 1 | 2 | 12 | 0.0007 | 0.0238 | 36.21 | group-specific protein |
| Cluster_4938 | 10 | 10 | 8 | 1 | 2 | 12 | 0.0007 | 0.0238 | 36.21 | hypothetical protein |
| Cluster_4942 | 10 | 10 | 8 | 1 | 2 | 12 | 0.0007 | 0.0238 | 36.21 | hypothetical protein |
| Cluster_4950 | 10 | 10 | 8 | 1 | 2 | 12 | 0.0007 | 0.0238 | 36.21 | hypothetical protein |
| Cluster_4951 | 10 | 10 | 8 | 1 | 2 | 12 | 0.0007 | 0.0238 | 36.21 | hypothetical protein |
| Cluster_4962 | 10 | 10 | 8 | 1 | 2 | 12 | 0.0007 | 0.0238 | 36.21 | hypothetical protein |
| Cluster_4970 | 10 | 10 | 8 | 1 | 2 | 12 | 0.0007 | 0.0238 | 36.21 | hypothetical protein |
| Cluster_4971 | 10 | 10 | 8 | 1 | 2 | 12 | 0.0007 | 0.0238 | 36.21 | hypothetical protein |
| Cluster_4974 | 10 | 10 | 8 | 1 | 2 | 12 | 0.0007 | 0.0238 | 36.21 | hypothetical protein |
| Cluster_4975 | 10 | 10 | 8 | 1 | 2 | 12 | 0.0007 | 0.0238 | 36.21 | hypothetical protein |
| Cluster_4976 | 10 | 10 | 8 | 1 | 2 | 12 | 0.0007 | 0.0238 | 36.21 | hypothetical protein |
| Cluster_4949 | 10 | 10 | 8 | 1 | 2 | 12 | 0.0007 | 0.0238 | 36.21 | methyltransferase |
| Cluster_4944 | 10 | 10 | 8 | 1 | 2 | 12 | 0.0007 | 0.0238 | 36.21 | molybdopterin synthase sulfur carrier subunit |
| Cluster_4936 | 10 | 10 | 8 | 1 | 2 | 12 | 0.0007 | 0.0238 | 36.21 | N-acetylmuramoyl-L-alanine amidase |
| Cluster_4918 | 10 | 10 | 8 | 1 | 2 | 12 | 0.0007 | 0.0238 | 36.21 | PbsX family transcriptional regulator |
| Cluster_4946 | 10 | 10 | 8 | 1 | 2 | 12 | 0.0007 | 0.0238 | 36.21 | S-layer protein |
| Cluster_4969 | 10 | 10 | 8 | 1 | 2 | 12 | 0.0007 | 0.0238 | 36.21 | TetR family transcriptional regulator |
| Cluster_4965 | 10 | 10 | 8 | 1 | 2 | 12 | 0.0007 | 0.0238 | 36.21 | thiamine pyrophosphate-binding protein |
| Cluster_4866 | 10 | 10 | 8 | 1 | 2 | 12 | 0.0007 | 0.0238 | 36.21 | transcriptional regulator |
| Cluster_4919 | 10 | 10 | 8 | 1 | 2 | 12 | 0.0007 | 0.0238 | 36.21 | Virginiamycin B lyase |
| Cluster_4754 | 11 | 11 | 9 | 0 | 2 | 12 | 6.73E-05 | 0.0053 | Inf | 6-phosphogluconolactonase |
| Cluster_4736 | 11 | 11 | 9 | 0 | 2 | 12 | 6.73E-05 | 0.0053 | Inf | ABC transporter permease |
| Cluster_4779 | 11 | 11 | 9 | 0 | 2 | 12 | 6.73E-05 | 0.0053 | Inf | ABC transporter permease |
| Cluster_4701 | 11 | 11 | 9 | 0 | 2 | 12 | 6.73E-05 | 0.0053 | Inf | acetyltransferase |
| Cluster_4717 | 11 | 11 | 9 | 0 | 2 | 12 | 6.73E-05 | 0.0053 | Inf | acetyltransferase |
| Cluster_4730 | 11 | 11 | 9 | 0 | 2 | 12 | 6.73E-05 | 0.0053 | Inf | acetyltransferase |
| Cluster_4768 | 11 | 11 | 9 | 0 | 2 | 12 | 6.73E-05 | 0.0053 | Inf | acetyltransferase |
| Cluster_4790 | 11 | 11 | 9 | 0 | 2 | 12 | 6.73E-05 | 0.0053 | Inf | acetyltransferase |
| Cluster_4711 | 11 | 11 | 9 | 0 | 2 | 12 | 6.73E-05 | 0.0053 | Inf | alkyl hydroperoxide reductase |
| Cluster_4703 | 11 | 11 | 9 | 0 | 2 | 12 | 6.73E-05 | 0.0053 | Inf | alpha/beta hydrolase |
| Cluster_4745 | 11 | 11 | 9 | 0 | 2 | 12 | 6.73E-05 | 0.0053 | Inf | aminoglycoside phosphotransferase |
| Cluster_4794 | 11 | 11 | 9 | 0 | 2 | 12 | 6.73E-05 | 0.0053 | Inf | aminoglycoside phosphotransferase |
| Cluster_4741 | 11 | 11 | 9 | 0 | 2 | 12 | 6.73E-05 | 0.0053 | Inf | antibiotic biosynthesis monooxygenase |
| Cluster_4739 | 11 | 11 | 9 | 0 | 2 | 12 | 6.73E-05 | 0.0053 | Inf | AraC family transcriptional regulator |
| Cluster_4793 | 11 | 11 | 9 | 0 | 2 | 12 | 6.73E-05 | 0.0053 | Inf | AraC family transcriptional regulator |
| Cluster_4803 | 11 | 11 | 9 | 0 | 2 | 12 | 6.73E-05 | 0.0053 | Inf | capsular biosynthesis protein |
| Cluster_4749 | 11 | 11 | 9 | 0 | 2 | 12 | 6.73E-05 | 0.0053 | Inf | cell surface protein |
| Cluster_4764 | 11 | 11 | 9 | 0 | 2 | 12 | 6.73E-05 | 0.0053 | Inf | cell wall anchor protein |
| Cluster_4778 | 11 | 11 | 9 | 0 | 2 | 12 | 6.73E-05 | 0.0053 | Inf | chromosome segregation protein |
| Cluster_4759 | 11 | 11 | 9 | 0 | 2 | 12 | 6.73E-05 | 0.0053 | Inf | competence protein ComF |
| Cluster_4731 | 11 | 11 | 9 | 0 | 2 | 12 | 6.73E-05 | 0.0053 | Inf | competence protein ComG |
| Cluster_4732 | 11 | 11 | 9 | 0 | 2 | 12 | 6.73E-05 | 0.0053 | Inf | competence protein ComG |
| Cluster_4733 | 11 | 11 | 9 | 0 | 2 | 12 | 6.73E-05 | 0.0053 | Inf | competence protein ComG |
| Cluster_4734 | 11 | 11 | 9 | 0 | 2 | 12 | 6.73E-05 | 0.0053 | Inf | competence protein ComG |
| Cluster_4719 | 11 | 11 | 9 | 0 | 2 | 12 | 6.73E-05 | 0.0053 | Inf | competence protein |
| Cluster_4756 | 11 | 11 | 9 | 0 | 2 | 12 | 6.73E-05 | 0.0053 | Inf | cytochrome C oxidase subunit II |
| Cluster_4781 | 11 | 11 | 9 | 0 | 2 | 12 | 6.73E-05 | 0.0053 | Inf | DNA mismatch repair protein MutT |
| Cluster_4735 | 11 | 11 | 9 | 0 | 2 | 12 | 6.73E-05 | 0.0053 | Inf | DNA recombination protein RecO |
| Cluster_4769 | 11 | 11 | 9 | 0 | 2 | 12 | 6.73E-05 | 0.0053 | Inf | glutamine amidotransferase |
| Cluster_4726 | 11 | 11 | 9 | 0 | 2 | 12 | 6.73E-05 | 0.0053 | Inf | glycosyl transferase |
| Cluster_4727 | 11 | 11 | 9 | 0 | 2 | 12 | 6.73E-05 | 0.0053 | Inf | glycosyl transferase |
| Cluster_4700 | 11 | 11 | 9 | 0 | 2 | 12 | 6.73E-05 | 0.0053 | Inf | glyoxalase |
| Cluster_4699 | 11 | 11 | 9 | 0 | 2 | 12 | 6.73E-05 | 0.0053 | Inf | GNAT family acetyltransferase |
| Cluster_4725 | 11 | 11 | 9 | 0 | 2 | 12 | 6.73E-05 | 0.0053 | Inf | histidine kinase |
| Cluster_4805 | 11 | 11 | 9 | 0 | 2 | 12 | 6.73E-05 | 0.0053 | Inf | histidine kinase |
| Cluster_4806 | 11 | 11 | 9 | 0 | 2 | 12 | 6.73E-05 | 0.0053 | Inf | histidine kinase |
| Cluster_4729 | 11 | 11 | 9 | 0 | 2 | 12 | 6.73E-05 | 0.0053 | Inf | histidine phosphatase family protein |
| Cluster_4358 | 13 | 11 | 9 | 0 | 2 | 12 | 6.73E-05 | 0.0053 | Inf | hypothetical protein |
| Cluster_4697 | 11 | 11 | 9 | 0 | 2 | 12 | 6.73E-05 | 0.0053 | Inf | hypothetical protein |
| Cluster_4698 | 11 | 11 | 9 | 0 | 2 | 12 | 6.73E-05 | 0.0053 | Inf | hypothetical protein |
| Cluster_4704 | 11 | 11 | 9 | 0 | 2 | 12 | 6.73E-05 | 0.0053 | Inf | hypothetical protein |
| Cluster_4705 | 11 | 11 | 9 | 0 | 2 | 12 | 6.73E-05 | 0.0053 | Inf | hypothetical protein |
| Cluster_4707 | 11 | 11 | 9 | 0 | 2 | 12 | 6.73E-05 | 0.0053 | Inf | hypothetical protein |
| Cluster_4709 | 11 | 11 | 9 | 0 | 2 | 12 | 6.73E-05 | 0.0053 | Inf | hypothetical protein |
| Cluster_4721 | 11 | 11 | 9 | 0 | 2 | 12 | 6.73E-05 | 0.0053 | Inf | hypothetical protein |
| Cluster_4728 | 11 | 11 | 9 | 0 | 2 | 12 | 6.73E-05 | 0.0053 | Inf | hypothetical protein |
| Cluster_4737 | 11 | 11 | 9 | 0 | 2 | 12 | 6.73E-05 | 0.0053 | Inf | hypothetical protein |
| Cluster_4740 | 11 | 11 | 9 | 0 | 2 | 12 | 6.73E-05 | 0.0053 | Inf | hypothetical protein |
| Cluster_4742 | 11 | 11 | 9 | 0 | 2 | 12 | 6.73E-05 | 0.0053 | Inf | hypothetical protein |
| Cluster_4743 | 11 | 11 | 9 | 0 | 2 | 12 | 6.73E-05 | 0.0053 | Inf | hypothetical protein |
| Cluster_4744 | 11 | 11 | 9 | 0 | 2 | 12 | 6.73E-05 | 0.0053 | Inf | hypothetical protein |
| Cluster_4746 | 11 | 11 | 9 | 0 | 2 | 12 | 6.73E-05 | 0.0053 | Inf | hypothetical protein |
| Cluster_4748 | 11 | 11 | 9 | 0 | 2 | 12 | 6.73E-05 | 0.0053 | Inf | hypothetical protein |
| Cluster_4750 | 11 | 11 | 9 | 0 | 2 | 12 | 6.73E-05 | 0.0053 | Inf | hypothetical protein |
| Cluster_4767 | 11 | 11 | 9 | 0 | 2 | 12 | 6.73E-05 | 0.0053 | Inf | hypothetical protein |
| Cluster_4782 | 11 | 11 | 9 | 0 | 2 | 12 | 6.73E-05 | 0.0053 | Inf | hypothetical protein |
| Cluster_4787 | 11 | 11 | 9 | 0 | 2 | 12 | 6.73E-05 | 0.0053 | Inf | hypothetical protein |
| Cluster_4791 | 11 | 11 | 9 | 0 | 2 | 12 | 6.73E-05 | 0.0053 | Inf | hypothetical protein |
| Cluster_4792 | 11 | 11 | 9 | 0 | 2 | 12 | 6.73E-05 | 0.0053 | Inf | hypothetical protein |
| Cluster_4797 | 11 | 11 | 9 | 0 | 2 | 12 | 6.73E-05 | 0.0053 | Inf | hypothetical protein |
| Cluster_4800 | 11 | 11 | 9 | 0 | 2 | 12 | 6.73E-05 | 0.0053 | Inf | hypothetical protein |
| Cluster_4801 | 11 | 11 | 9 | 0 | 2 | 12 | 6.73E-05 | 0.0053 | Inf | hypothetical protein |
| Cluster_4802 | 11 | 11 | 9 | 0 | 2 | 12 | 6.73E-05 | 0.0053 | Inf | hypothetical protein |
| Cluster_4804 | 11 | 11 | 9 | 0 | 2 | 12 | 6.73E-05 | 0.0053 | Inf | hypothetical protein |
| Cluster_4808 | 11 | 11 | 9 | 0 | 2 | 12 | 6.73E-05 | 0.0053 | Inf | hypothetical protein |
| Cluster_4973 | 10 | 10 | 9 | 0 | 1 | 13 | 1.22E-05 | 0.0053 | Inf | hypothetical protein |
| Cluster_5006 | 10 | 10 | 9 | 0 | 1 | 13 | 1.22E-05 | 0.0053 | Inf | hypothetical protein |
| Cluster_5007 | 10 | 10 | 9 | 0 | 1 | 13 | 1.22E-05 | 0.0053 | Inf | hypothetical protein |
| Cluster_5010 | 10 | 10 | 9 | 0 | 1 | 13 | 1.22E-05 | 0.0053 | Inf | hypothetical protein |
| Cluster_4691 | 11 | 11 | 9 | 0 | 2 | 12 | 6.73E-05 | 0.0053 | Inf | invasion protein |
| Cluster_4799 | 11 | 11 | 9 | 0 | 2 | 12 | 6.73E-05 | 0.0053 | Inf | invasion protein |
| Cluster_4718 | 11 | 11 | 9 | 0 | 2 | 12 | 6.73E-05 | 0.0053 | Inf | LacI family transcriptional regulator |
| Cluster_4770 | 11 | 11 | 9 | 0 | 2 | 12 | 6.73E-05 | 0.0053 | Inf | LysR family transcriptional regulator |
| Cluster_4738 | 11 | 11 | 9 | 0 | 2 | 12 | 6.73E-05 | 0.0053 | Inf | MepB |
| Cluster_4788 | 11 | 11 | 9 | 0 | 2 | 12 | 6.73E-05 | 0.0053 | Inf | methyltransferase |
| Cluster_4796 | 11 | 11 | 9 | 0 | 2 | 12 | 6.73E-05 | 0.0053 | Inf | PadR family transcriptional regulator |
| Cluster_4761 | 11 | 11 | 9 | 0 | 2 | 12 | 6.73E-05 | 0.0053 | Inf | peptidase G2 |
| Cluster_4708 | 11 | 11 | 9 | 0 | 2 | 12 | 6.73E-05 | 0.0053 | Inf | peptide transporter |
| Cluster_4798 | 11 | 11 | 9 | 0 | 2 | 12 | 6.73E-05 | 0.0053 | Inf | peptide-binding protein |
| Cluster_4809 | 11 | 11 | 9 | 0 | 2 | 12 | 6.73E-05 | 0.0053 | Inf | permease |
| Cluster_4771 | 11 | 11 | 9 | 0 | 2 | 12 | 6.73E-05 | 0.0053 | Inf | phage tail protein |
| Cluster_4807 | 11 | 11 | 9 | 0 | 2 | 12 | 6.73E-05 | 0.0053 | Inf | phosphoglycerol transferase |
| Cluster_4722 | 11 | 11 | 9 | 0 | 2 | 12 | 6.73E-05 | 0.0053 | Inf | preprotein translocase |
| Cluster_4710 | 11 | 11 | 9 | 0 | 2 | 12 | 6.73E-05 | 0.0053 | Inf | ROK family transcriptional regulator |
| Cluster_5008 | 10 | 10 | 9 | 0 | 1 | 13 | 1.22E-05 | 0.0053 | Inf | serine hydrolase |
| Cluster_4963 | 10 | 10 | 9 | 0 | 1 | 13 | 1.22E-05 | 0.0053 | Inf | serine/threonine protein kinase |
| Cluster_4702 | 11 | 11 | 9 | 0 | 2 | 12 | 6.73E-05 | 0.0053 | Inf | siderophore biosynthesis protein |
| Cluster_4389 | 13 | 11 | 9 | 0 | 2 | 12 | 6.73E-05 | 0.0053 | Inf | sporulation protein |
| Cluster_4789 | 11 | 11 | 9 | 0 | 2 | 12 | 6.73E-05 | 0.0053 | Inf | sporulation protein |
| Cluster_4832 | 11 | 11 | 9 | 0 | 2 | 12 | 6.73E-05 | 0.0053 | Inf | sugar ABC transporter ATP-binding protein |
| Cluster_4692 | 11 | 11 | 9 | 0 | 2 | 12 | 6.73E-05 | 0.0053 | Inf | teicoplanin resistance protein VanZ |
| Cluster_4943 | 10 | 10 | 9 | 0 | 1 | 13 | 1.22E-05 | 0.0053 | Inf | transglycosylase |
| Cluster_4367 | 13 | 11 | 9 | 0 | 2 | 12 | 6.73E-05 | 0.0053 | Inf | translation initiation inhibitor |
| Cluster_4706 | 11 | 11 | 9 | 0 | 2 | 12 | 6.73E-05 | 0.0053 | Inf | uridine kinase |
| Cluster_4510 | 12 | 12 | 9 | 0 | 3 | 11 | 0.0003 | 0.0126 | Inf | 3-phosphoshikimate 1-carboxyvinyltransferase |
| Cluster_4518 | 12 | 12 | 9 | 0 | 3 | 11 | 0.0003 | 0.0126 | Inf | 4-hydroxy-2-ketovalerate aldolase |
| Cluster_4533 | 12 | 12 | 9 | 0 | 3 | 11 | 0.0003 | 0.0126 | Inf | ABC transporter |
| Cluster_4541 | 12 | 12 | 9 | 0 | 3 | 11 | 0.0003 | 0.0126 | Inf | ABC transporter |
| Cluster_4523 | 12 | 12 | 9 | 0 | 3 | 11 | 0.0003 | 0.0126 | Inf | alkaline phosphatase |
| Cluster_4449 | 12 | 12 | 9 | 0 | 3 | 11 | 0.0003 | 0.0126 | Inf | alpha/beta hydrolase |
| Cluster_4507 | 12 | 12 | 9 | 0 | 3 | 11 | 0.0003 | 0.0126 | Inf | betaine-aldehyde dehydrogenase |
| Cluster_4457 | 12 | 12 | 9 | 0 | 3 | 11 | 0.0003 | 0.0126 | Inf | damage-inducible protein DinB |
| Cluster_4511 | 12 | 12 | 9 | 0 | 3 | 11 | 0.0003 | 0.0126 | Inf | DNA helicase |
| Cluster_4450 | 12 | 12 | 9 | 0 | 3 | 11 | 0.0003 | 0.0126 | Inf | DNA-binding response regulator |
| Cluster_4529 | 12 | 12 | 9 | 0 | 3 | 11 | 0.0003 | 0.0126 | Inf | epimerase |
| Cluster_4506 | 12 | 12 | 9 | 0 | 3 | 11 | 0.0003 | 0.0126 | Inf | Fis family transcriptional regulator |
| Cluster_4526 | 12 | 12 | 9 | 0 | 3 | 11 | 0.0003 | 0.0126 | Inf | GntR family transcriptional regulator |
| Cluster_4432 | 12 | 12 | 9 | 0 | 3 | 11 | 0.0003 | 0.0126 | Inf | hypothetical protein |
| Cluster_4505 | 12 | 12 | 9 | 0 | 3 | 11 | 0.0003 | 0.0126 | Inf | hypothetical protein |
| Cluster_4509 | 12 | 12 | 9 | 0 | 3 | 11 | 0.0003 | 0.0126 | Inf | hypothetical protein |
| Cluster_4522 | 12 | 12 | 9 | 0 | 3 | 11 | 0.0003 | 0.0126 | Inf | hypothetical protein |
| Cluster_4537 | 12 | 12 | 9 | 0 | 3 | 11 | 0.0003 | 0.0126 | Inf | hypothetical protein |
| Cluster_4530 | 12 | 12 | 9 | 0 | 3 | 11 | 0.0003 | 0.0126 | Inf | MBL fold metallo-hydrolase |
| Cluster_4527 | 12 | 12 | 9 | 0 | 3 | 11 | 0.0003 | 0.0126 | Inf | MFS transporter |
| Cluster_4536 | 12 | 12 | 9 | 0 | 3 | 11 | 0.0003 | 0.0126 | Inf | MFS transporter |
| Cluster_4539 | 12 | 12 | 9 | 0 | 3 | 11 | 0.0003 | 0.0126 | Inf | MFS transporter |
| Cluster_4532 | 12 | 12 | 9 | 0 | 3 | 11 | 0.0003 | 0.0126 | Inf | multidrug ABC transporter ATP-binding protein |
| Cluster_4508 | 12 | 12 | 9 | 0 | 3 | 11 | 0.0003 | 0.0126 | Inf | putrescine aminotransferase |
| Cluster_4504 | 12 | 12 | 9 | 0 | 3 | 11 | 0.0003 | 0.0126 | Inf | Putrescine importer PuuP |
| Cluster_4535 | 12 | 12 | 9 | 0 | 3 | 11 | 0.0003 | 0.0126 | Inf | short-chain dehydrogenase |
| Cluster_4500 | 12 | 12 | 9 | 0 | 3 | 11 | 0.0003 | 0.0126 | Inf | sporulation protein |
| Cluster_4370 | 13 | 13 | 9 | 0 | 4 | 10 | 0.0016 | 0.0411 | Inf | ABC transporter ATP-binding protein |
| Cluster_4377 | 13 | 13 | 9 | 0 | 4 | 10 | 0.0016 | 0.0411 | Inf | amino acid permease |
| Cluster_4336 | 13 | 13 | 9 | 0 | 4 | 10 | 0.0016 | 0.0411 | Inf | cyclic nucleotide-binding protein |
| Cluster_4407 | 13 | 13 | 9 | 0 | 4 | 10 | 0.0016 | 0.0411 | Inf | cysteine ABC transporter permease |
| Cluster_4213 | 15 | 13 | 9 | 0 | 4 | 10 | 0.0016 | 0.0412 | Inf | DUF3948 domain-containing protein |
| Cluster_4379 | 13 | 13 | 9 | 0 | 4 | 10 | 0.0016 | 0.0411 | Inf | Fe-S oxidoreductase |
| Cluster_4380 | 13 | 13 | 9 | 0 | 4 | 10 | 0.0016 | 0.0411 | Inf | hydroxyglutarate oxidase |
| Cluster_4345 | 13 | 13 | 9 | 0 | 4 | 10 | 0.0016 | 0.0411 | Inf | hypothetical protein |
| Cluster_4375 | 13 | 13 | 9 | 0 | 4 | 10 | 0.0016 | 0.0411 | Inf | hypothetical protein |
| Cluster_4376 | 13 | 13 | 9 | 0 | 4 | 10 | 0.0016 | 0.0411 | Inf | hypothetical protein |
| Cluster_4388 | 13 | 13 | 9 | 0 | 4 | 10 | 0.0016 | 0.0411 | Inf | hypothetical protein |
| Cluster_4378 | 13 | 13 | 9 | 0 | 4 | 10 | 0.0016 | 0.0411 | Inf | lactate utilization protein C |
| Cluster_4363 | 13 | 13 | 9 | 0 | 4 | 10 | 0.0016 | 0.0411 | Inf | NADPH:quinone oxidoreductase |
| Cluster_4382 | 13 | 13 | 9 | 0 | 4 | 10 | 0.0016 | 0.0411 | Inf | ornithine cyclodeaminase |
| Cluster_4381 | 13 | 13 | 9 | 0 | 4 | 10 | 0.0016 | 0.0411 | Inf | protein CsiD |
| Cluster_4409 | 13 | 13 | 9 | 0 | 4 | 10 | 0.0016 | 0.0411 | Inf | ribonucleotide-diphosphate reductase subunit |
| Cluster_4371 | 13 | 13 | 9 | 0 | 4 | 10 | 0.0016 | 0.0411 | Inf | spore coat protein |
| Cluster_4359 | 13 | 13 | 9 | 0 | 4 | 10 | 0.0016 | 0.0411 | Inf | stage II sporulation protein P |

*^a^* P-values from two-sided Fisher's Exact Tests

*^b^* P-values were corrected using the False Discovery Rate (FDR)

*^c^* Odds ratios marked as INF (Infinite) are a result of dividing by zero
