# Supplementary material for: Genes Associated With Psychrotolerant Bacillus cereus Group Isolates
Source: Front Microbiol. 2019 Mar 29;10:662. doi: 10.3389/fmicb.2019.00662 (PMC6449464; doi:10.3389/fmicb.2019.00662)
Supplement: Supplementary file 4 [file Table_4.DOCX]

**Supplemental Table 4**: OrthoMCL clusters: List of gene clusters encoded in the genomes of non-psychrotolerant (< 1log_10_ increase after 21-day incubation at 6°C in BHI broth) *B. cereus* group isolates

| Cluster | Number of Genes | Number of Taxa | Presence among Psychrot-olerant Strains | Absence among Psychr-otolerant Strains | Presence among Non-Psychrot-olerant Strains | Absence among Non-Psychrot-olerant Strains | P-value*^a^* | FDR*^b^* | Odds Ratio*^c^* | Products |
| --- | --- | --- | --- | --- | --- | --- | --- | --- | --- | --- |
| Cluster_4415 | 12 | 12 | 0 | 9 | 12 | 2 | 6.73E-05 | 0.0053 | 0 | 50S ribosomal protein L33 |
| Cluster_4465 | 12 | 12 | 0 | 9 | 12 | 2 | 6.73E-05 | 0.0053 | 0 | ferrochelatase |
| Cluster_4453 | 12 | 12 | 0 | 9 | 12 | 2 | 6.73E-05 | 0.0053 | 0 | HAD family hydrolase |
| Cluster_4222 | 14 | 13 | 0 | 9 | 13 | 1 | 1.22E-05 | 0.0053 | 0 | hypothetical protein |
| Cluster_4298 | 13 | 13 | 0 | 9 | 13 | 1 | 1.22E-05 | 0.0053 | 0 | hypothetical protein |
| Cluster_4300 | 13 | 13 | 0 | 9 | 13 | 1 | 1.22E-05 | 0.0053 | 0 | hypothetical protein |
| Cluster_4301 | 13 | 13 | 0 | 9 | 13 | 1 | 1.22E-05 | 0.0053 | 0 | hypothetical protein |
| Cluster_4304 | 13 | 13 | 0 | 9 | 13 | 1 | 1.22E-05 | 0.0053 | 0 | hypothetical protein |
| Cluster_4309 | 13 | 12 | 0 | 9 | 12 | 2 | 6.73E-05 | 0.0053 | 0 | hypothetical protein |
| Cluster_4314 | 13 | 13 | 0 | 9 | 13 | 1 | 1.22E-05 | 0.0053 | 0 | hypothetical protein |
| Cluster_4319 | 13 | 13 | 0 | 9 | 13 | 1 | 1.22E-05 | 0.0053 | 0 | hypothetical protein |
| Cluster_4320 | 13 | 13 | 0 | 9 | 13 | 1 | 1.22E-05 | 0.0053 | 0 | hypothetical protein |
| Cluster_4328 | 13 | 13 | 0 | 9 | 13 | 1 | 1.22E-05 | 0.0053 | 0 | hypothetical protein |
| Cluster_4329 | 13 | 13 | 0 | 9 | 13 | 1 | 1.22E-05 | 0.0053 | 0 | hypothetical protein |
| Cluster_4335 | 13 | 13 | 0 | 9 | 13 | 1 | 1.22E-05 | 0.0053 | 0 | hypothetical protein |
| Cluster_4339 | 13 | 13 | 0 | 9 | 13 | 1 | 1.22E-05 | 0.0053 | 0 | hypothetical protein |
| Cluster_4350 | 13 | 13 | 0 | 9 | 13 | 1 | 1.22E-05 | 0.0053 | 0 | hypothetical protein |
| Cluster_4355 | 13 | 13 | 0 | 9 | 13 | 1 | 1.22E-05 | 0.0053 | 0 | hypothetical protein |
| Cluster_4364 | 13 | 12 | 0 | 9 | 12 | 2 | 6.73E-05 | 0.0053 | 0 | hypothetical protein |
| Cluster_4461 | 12 | 12 | 0 | 9 | 12 | 2 | 6.73E-05 | 0.0053 | 0 | hypothetical protein |
| Cluster_4462 | 12 | 12 | 0 | 9 | 12 | 2 | 6.73E-05 | 0.0053 | 0 | hypothetical protein |
| Cluster_4463 | 12 | 12 | 0 | 9 | 12 | 2 | 6.73E-05 | 0.0053 | 0 | hypothetical protein |
| Cluster_4473 | 12 | 12 | 0 | 9 | 12 | 2 | 6.73E-05 | 0.0053 | 0 | hypothetical protein |
| Cluster_4474 | 12 | 12 | 0 | 9 | 12 | 2 | 6.73E-05 | 0.0053 | 0 | hypothetical protein |
| Cluster_4490 | 12 | 12 | 0 | 9 | 12 | 2 | 6.73E-05 | 0.0053 | 0 | hypothetical protein |
| Cluster_4494 | 12 | 12 | 0 | 9 | 12 | 2 | 6.73E-05 | 0.0053 | 0 | hypothetical protein |
| Cluster_4515 | 12 | 12 | 0 | 9 | 12 | 2 | 6.73E-05 | 0.0053 | 0 | hypothetical protein |
| Cluster_4524 | 12 | 12 | 0 | 9 | 12 | 2 | 6.73E-05 | 0.0053 | 0 | hypothetical protein |
| Cluster_4531 | 12 | 12 | 0 | 9 | 12 | 2 | 6.73E-05 | 0.0053 | 0 | hypothetical protein |
| Cluster_4413 | 12 | 12 | 0 | 9 | 12 | 2 | 6.73E-05 | 0.0053 | 0 | transporter |
| Cluster_4434 | 12 | 12 | 0 | 9 | 12 | 2 | 6.73E-05 | 0.0053 | 0 | ubiquinone biosynthesis methyltransferase UbiE |
| Cluster_4452 | 12 | 12 | 0 | 9 | 12 | 2 | 6.73E-05 | 0.0053 | 0 | XRE family transcriptional regulator |
| Cluster_4437 | 12 | 11 | 0 | 9 | 11 | 3 | 0.0003 | 0.0126 | 0 | 2'-5' RNA ligase |
| Cluster_4590 | 11 | 11 | 0 | 9 | 11 | 3 | 0.0003 | 0.0126 | 0 | ABC transporter ATP-binding protein |
| Cluster_4615 | 11 | 11 | 0 | 9 | 11 | 3 | 0.0003 | 0.0126 | 0 | ABC transporter ATP-binding protein |
| Cluster_4477 | 12 | 11 | 0 | 9 | 11 | 3 | 0.0003 | 0.0126 | 0 | ABC transporter permease |
| Cluster_4649 | 11 | 11 | 0 | 9 | 11 | 3 | 0.0003 | 0.0126 | 0 | ABC transporter permease |
| Cluster_4624 | 11 | 11 | 0 | 9 | 11 | 3 | 0.0003 | 0.0126 | 0 | acetyl-CoA hydrolase |
| Cluster_4416 | 12 | 11 | 0 | 9 | 11 | 3 | 0.0003 | 0.0126 | 0 | acetyltransferases |
| Cluster_4571 | 11 | 11 | 0 | 9 | 11 | 3 | 0.0003 | 0.0126 | 0 | acetyltransferase |
| Cluster_4677 | 11 | 11 | 0 | 9 | 11 | 3 | 0.0003 | 0.0126 | 0 | acetyltransferase |
| Cluster_4683 | 11 | 11 | 0 | 9 | 11 | 3 | 0.0003 | 0.0126 | 0 | acetyltransferase |
| Cluster_4604 | 11 | 11 | 0 | 9 | 11 | 3 | 0.0003 | 0.0126 | 0 | alkaline phosphatase |
| Cluster_4438 | 12 | 11 | 0 | 9 | 11 | 3 | 0.0003 | 0.0126 | 0 | alkanesulfonate monooxygenase |
| Cluster_4657 | 11 | 11 | 0 | 9 | 11 | 3 | 0.0003 | 0.0126 | 0 | alpha/beta hydrolase |
| Cluster_4682 | 11 | 11 | 0 | 9 | 11 | 3 | 0.0003 | 0.0126 | 0 | alpha/beta hydrolase |
| Cluster_4631 | 11 | 11 | 0 | 9 | 11 | 3 | 0.0003 | 0.0126 | 0 | aminoglycoside phosphotransferase |
| Cluster_4653 | 11 | 11 | 0 | 9 | 11 | 3 | 0.0003 | 0.0126 | 0 | antibiotic biosynthesis monooxygenase |
| Cluster_4476 | 12 | 11 | 0 | 9 | 11 | 3 | 0.0003 | 0.0126 | 0 | AraC family transcriptional regulator |
| Cluster_4585 | 11 | 11 | 0 | 9 | 11 | 3 | 0.0003 | 0.0126 | 0 | AraC family transcriptional regulator |
| Cluster_4628 | 11 | 11 | 0 | 9 | 11 | 3 | 0.0003 | 0.0126 | 0 | cell surface protein" |
| Cluster_4650 | 11 | 11 | 0 | 9 | 11 | 3 | 0.0003 | 0.0126 | 0 | chromosome segregation protein |
| Cluster_4578 | 11 | 11 | 0 | 9 | 11 | 3 | 0.0003 | 0.0126 | 0 | collagenase |
| Cluster_4619 | 11 | 11 | 0 | 9 | 11 | 3 | 0.0003 | 0.0126 | 0 | competence protein ComF |
| Cluster_4573 | 11 | 11 | 0 | 9 | 11 | 3 | 0.0003 | 0.0126 | 0 | competence protein ComG |
| Cluster_4574 | 11 | 11 | 0 | 9 | 11 | 3 | 0.0003 | 0.0126 | 0 | competence protein ComG |
| Cluster_4575 | 11 | 11 | 0 | 9 | 11 | 3 | 0.0003 | 0.0126 | 0 | competence protein ComG |
| Cluster_4576 | 11 | 11 | 0 | 9 | 11 | 3 | 0.0003 | 0.0126 | 0 | competence protein |
| Cluster_4618 | 11 | 11 | 0 | 9 | 11 | 3 | 0.0003 | 0.0126 | 0 | competence protein |
| Cluster_4629 | 11 | 11 | 0 | 9 | 11 | 3 | 0.0003 | 0.0126 | 0 | cytochrome B |
| Cluster_4570 | 11 | 11 | 0 | 9 | 11 | 3 | 0.0003 | 0.0126 | 0 | delta-aminolevulinic acid dehydratase |
| Cluster_4642 | 11 | 11 | 0 | 9 | 11 | 3 | 0.0003 | 0.0126 | 0 | DNA mismatch repair protein MutT |
| Cluster_4658 | 11 | 11 | 0 | 9 | 11 | 3 | 0.0003 | 0.0126 | 0 | DNA-binding protein |
| Cluster_4640 | 11 | 11 | 0 | 9 | 11 | 3 | 0.0003 | 0.0126 | 0 | glycerophosphodiester phosphodiesterase |
| Cluster_4622 | 11 | 11 | 0 | 9 | 11 | 3 | 0.0003 | 0.0126 | 0 | glycosyl transferase family 2 |
| Cluster_4659 | 11 | 11 | 0 | 9 | 11 | 3 | 0.0003 | 0.0126 | 0 | glycosyl transferase |
| Cluster_4572 | 11 | 11 | 0 | 9 | 11 | 3 | 0.0003 | 0.0126 | 0 | group-specific protein |
| Cluster_4644 | 11 | 11 | 0 | 9 | 11 | 3 | 0.0003 | 0.0126 | 0 | group-specific protein |
| Cluster_4564 | 11 | 11 | 0 | 9 | 11 | 3 | 0.0003 | 0.0126 | 0 | histidine kinase |
| Cluster_4652 | 11 | 11 | 0 | 9 | 11 | 3 | 0.0003 | 0.0126 | 0 | histidine kinase |
| Cluster_4669 | 11 | 11 | 0 | 9 | 11 | 3 | 0.0003 | 0.0126 | 0 | histidine kinase |
| Cluster_4569 | 11 | 11 | 0 | 9 | 11 | 3 | 0.0003 | 0.0126 | 0 | histidine phosphatase family protein |
| Cluster_4436 | 12 | 11 | 0 | 9 | 11 | 3 | 0.0003 | 0.0126 | 0 | hypothetical protein |
| Cluster_4566 | 11 | 11 | 0 | 9 | 11 | 3 | 0.0003 | 0.0126 | 0 | hypothetical protein |
| Cluster_4568 | 11 | 11 | 0 | 9 | 11 | 3 | 0.0003 | 0.0126 | 0 | hypothetical protein |
| Cluster_4592 | 11 | 11 | 0 | 9 | 11 | 3 | 0.0003 | 0.0126 | 0 | hypothetical protein |
| Cluster_4596 | 11 | 11 | 0 | 9 | 11 | 3 | 0.0003 | 0.0126 | 0 | hypothetical protein |
| Cluster_4598 | 11 | 11 | 0 | 9 | 11 | 3 | 0.0003 | 0.0126 | 0 | hypothetical protein |
| Cluster_4602 | 11 | 11 | 0 | 9 | 11 | 3 | 0.0003 | 0.0126 | 0 | hypothetical protein |
| Cluster_4610 | 11 | 11 | 0 | 9 | 11 | 3 | 0.0003 | 0.0126 | 0 | hypothetical protein |
| Cluster_4614 | 11 | 11 | 0 | 9 | 11 | 3 | 0.0003 | 0.0126 | 0 | hypothetical protein |
| Cluster_4623 | 11 | 11 | 0 | 9 | 11 | 3 | 0.0003 | 0.0126 | 0 | hypothetical protein |
| Cluster_4627 | 11 | 11 | 0 | 9 | 11 | 3 | 0.0003 | 0.0126 | 0 | hypothetical protein |
| Cluster_4630 | 11 | 11 | 0 | 9 | 11 | 3 | 0.0003 | 0.0126 | 0 | hypothetical protein |
| Cluster_4632 | 11 | 11 | 0 | 9 | 11 | 3 | 0.0003 | 0.0126 | 0 | hypothetical protein |
| Cluster_4633 | 11 | 11 | 0 | 9 | 11 | 3 | 0.0003 | 0.0126 | 0 | hypothetical protein |
| Cluster_4641 | 11 | 11 | 0 | 9 | 11 | 3 | 0.0003 | 0.0126 | 0 | hypothetical protein |
| Cluster_4643 | 11 | 11 | 0 | 9 | 11 | 3 | 0.0003 | 0.0126 | 0 | hypothetical protein |
| Cluster_4651 | 11 | 11 | 0 | 9 | 11 | 3 | 0.0003 | 0.0126 | 0 | hypothetical protein |
| Cluster_4654 | 11 | 11 | 0 | 9 | 11 | 3 | 0.0003 | 0.0126 | 0 | hypothetical protein |
| Cluster_4655 | 11 | 11 | 0 | 9 | 11 | 3 | 0.0003 | 0.0126 | 0 | hypothetical protein |
| Cluster_4656 | 11 | 11 | 0 | 9 | 11 | 3 | 0.0003 | 0.0126 | 0 | hypothetical protein |
| Cluster_4671 | 11 | 11 | 0 | 9 | 11 | 3 | 0.0003 | 0.0126 | 0 | hypothetical protein |
| Cluster_4680 | 11 | 11 | 0 | 9 | 11 | 3 | 0.0003 | 0.0126 | 0 | hypothetical protein |
| Cluster_4688 | 11 | 11 | 0 | 9 | 11 | 3 | 0.0003 | 0.0126 | 0 | hypothetical protein |
| Cluster_4712 | 11 | 11 | 0 | 9 | 11 | 3 | 0.0003 | 0.0126 | 0 | hypothetical protein |
| Cluster_4716 | 11 | 11 | 0 | 9 | 11 | 3 | 0.0003 | 0.0126 | 0 | hypothetical protein |
| Cluster_4720 | 11 | 11 | 0 | 9 | 11 | 3 | 0.0003 | 0.0126 | 0 | hypothetical protein |
| Cluster_4724 | 11 | 11 | 0 | 9 | 11 | 3 | 0.0003 | 0.0126 | 0 | hypothetical protein |
| Cluster_4752 | 11 | 11 | 0 | 9 | 11 | 3 | 0.0003 | 0.0126 | 0 | hypothetical protein |
| Cluster_4753 | 11 | 11 | 0 | 9 | 11 | 3 | 0.0003 | 0.0126 | 0 | hypothetical protein |
| Cluster_4758 | 11 | 11 | 0 | 9 | 11 | 3 | 0.0003 | 0.0126 | 0 | hypothetical protein |
| Cluster_4760 | 11 | 11 | 0 | 9 | 11 | 3 | 0.0003 | 0.0126 | 0 | hypothetical protein |
| Cluster_4765 | 11 | 11 | 0 | 9 | 11 | 3 | 0.0003 | 0.0126 | 0 | hypothetical protein |
| Cluster_4783 | 11 | 11 | 0 | 9 | 11 | 3 | 0.0003 | 0.0126 | 0 | hypothetical protein |
| Cluster_4810 | 11 | 11 | 0 | 9 | 11 | 3 | 0.0003 | 0.0126 | 0 | hypothetical protein |
| Cluster_4815 | 11 | 11 | 0 | 9 | 11 | 3 | 0.0003 | 0.0126 | 0 | hypothetical protein |
| Cluster_4417 | 12 | 11 | 0 | 9 | 11 | 3 | 0.0003 | 0.0126 | 0 | LacI family transcriptional regulator |
| Cluster_4625 | 11 | 11 | 0 | 9 | 11 | 3 | 0.0003 | 0.0126 | 0 | LytR family transcriptional regulator |
| Cluster_4608 | 11 | 11 | 0 | 9 | 11 | 3 | 0.0003 | 0.0126 | 0 | magnesium transporter |
| Cluster_4595 | 11 | 11 | 0 | 9 | 11 | 3 | 0.0003 | 0.0126 | 0 | mep operon protein MepB |
| Cluster_4679 | 11 | 11 | 0 | 9 | 11 | 3 | 0.0003 | 0.0126 | 0 | metal-dependent hydrolase |
| Cluster_4587 | 11 | 11 | 0 | 9 | 11 | 3 | 0.0003 | 0.0126 | 0 | methyltransferase |
| Cluster_4588 | 11 | 11 | 0 | 9 | 11 | 3 | 0.0003 | 0.0126 | 0 | methyltransferase |
| Cluster_4597 | 11 | 11 | 0 | 9 | 11 | 3 | 0.0003 | 0.0126 | 0 | MFS transporter |
| Cluster_4645 | 11 | 11 | 0 | 9 | 11 | 3 | 0.0003 | 0.0126 | 0 | molybdopterin synthase sulfur carrier subunit |
| Cluster_4635 | 11 | 11 | 0 | 9 | 11 | 3 | 0.0003 | 0.0126 | 0 | peptidase G2 |
| Cluster_4607 | 11 | 11 | 0 | 9 | 11 | 3 | 0.0003 | 0.0126 | 0 | phenazine biosynthesis protein PhzF |
| Cluster_4670 | 11 | 11 | 0 | 9 | 11 | 3 | 0.0003 | 0.0126 | 0 | rhodanese |
| Cluster_4617 | 11 | 11 | 0 | 9 | 11 | 3 | 0.0003 | 0.0126 | 0 | RNA polymerase subunit sigma-70 |
| Cluster_4591 | 11 | 11 | 0 | 9 | 11 | 3 | 0.0003 | 0.0126 | 0 | serine/threonine protein kinase |
| Cluster_4647 | 11 | 11 | 0 | 9 | 11 | 3 | 0.0003 | 0.0126 | 0 | spore gernimation protein |
| Cluster_4426 | 12 | 11 | 0 | 9 | 11 | 3 | 0.0003 | 0.0126 | 0 | sporulation protein |
| Cluster_4621 | 11 | 11 | 0 | 9 | 11 | 3 | 0.0003 | 0.0126 | 0 | stage II sporulation protein E |
| Cluster_4603 | 11 | 11 | 0 | 9 | 11 | 3 | 0.0003 | 0.0126 | 0 | sugar ABC transporter ATP-binding protein |
| Cluster_4439 | 12 | 11 | 0 | 9 | 11 | 3 | 0.0003 | 0.0126 | 0 | sulfonate ABC transporter permease |
| Cluster_4440 | 12 | 11 | 0 | 9 | 11 | 3 | 0.0003 | 0.0126 | 0 | sulfonate ABC transporter substrate-binding |
| Cluster_4589 | 11 | 11 | 0 | 9 | 11 | 3 | 0.0003 | 0.0126 | 0 | translation initiation inhibitor |
| Cluster_4418 | 12 | 11 | 0 | 9 | 11 | 3 | 0.0003 | 0.0126 | 0 | transporter |
| Cluster_4563 | 11 | 11 | 0 | 9 | 11 | 3 | 0.0003 | 0.0126 | 0 | two-component sensor histidine kinase |
| Cluster_4605 | 11 | 11 | 0 | 9 | 11 | 3 | 0.0003 | 0.0126 | 0 | uridine kinase |
| Cluster_4577 | 11 | 11 | 0 | 9 | 11 | 3 | 0.0003 | 0.0126 | 0 | YlmC/YmxH family sporulation protein |
| Cluster_4594 | 11 | 10 | 0 | 9 | 10 | 4 | 0.0016 | 0.0411 | 0 | acetyltransferase |
| Cluster_4601 | 11 | 10 | 0 | 9 | 10 | 4 | 0.0016 | 0.0411 | 0 | acetyltransferase |
| Cluster_4878 | 10 | 10 | 0 | 9 | 10 | 4 | 0.0016 | 0.0411 | 0 | acetyltransferase |
| Cluster_4899 | 10 | 10 | 0 | 9 | 10 | 4 | 0.0016 | 0.0411 | 0 | alkaline serine protease |
| Cluster_4992 | 10 | 10 | 0 | 9 | 10 | 4 | 0.0016 | 0.0411 | 0 | aminoglycoside phosphotransferase |
| Cluster_4896 | 10 | 10 | 0 | 9 | 10 | 4 | 0.0016 | 0.0411 | 0 | ATP-binding protein |
| Cluster_4835 | 10 | 10 | 0 | 9 | 10 | 4 | 0.0016 | 0.0411 | 0 | cell division protein FtsN |
| Cluster_4870 | 10 | 10 | 0 | 9 | 10 | 4 | 0.0016 | 0.0411 | 0 | cell wall anchor protein |
| Cluster_4995 | 10 | 10 | 0 | 9 | 10 | 4 | 0.0016 | 0.0411 | 0 | DNA recombination protein RecO |
| Cluster_4869 | 10 | 10 | 0 | 9 | 10 | 4 | 0.0016 | 0.0411 | 0 | group-specific protein |
| Cluster_4841 | 10 | 10 | 0 | 9 | 10 | 4 | 0.0016 | 0.0411 | 0 | hypothetical protein |
| Cluster_4843 | 10 | 10 | 0 | 9 | 10 | 4 | 0.0016 | 0.0411 | 0 | hypothetical protein |
| Cluster_4852 | 10 | 10 | 0 | 9 | 10 | 4 | 0.0016 | 0.0411 | 0 | hypothetical protein |
| Cluster_4862 | 10 | 10 | 0 | 9 | 10 | 4 | 0.0016 | 0.0411 | 0 | hypothetical protein |
| Cluster_4865 | 10 | 10 | 0 | 9 | 10 | 4 | 0.0016 | 0.0411 | 0 | hypothetical protein |
| Cluster_4874 | 10 | 10 | 0 | 9 | 10 | 4 | 0.0016 | 0.0411 | 0 | hypothetical protein |
| Cluster_4889 | 10 | 10 | 0 | 9 | 10 | 4 | 0.0016 | 0.0411 | 0 | hypothetical protein |
| Cluster_4891 | 10 | 10 | 0 | 9 | 10 | 4 | 0.0016 | 0.0411 | 0 | hypothetical protein |
| Cluster_4898 | 10 | 10 | 0 | 9 | 10 | 4 | 0.0016 | 0.0411 | 0 | hypothetical protein |
| Cluster_4904 | 10 | 10 | 0 | 9 | 10 | 4 | 0.0016 | 0.0411 | 0 | hypothetical protein |
| Cluster_4905 | 10 | 10 | 0 | 9 | 10 | 4 | 0.0016 | 0.0411 | 0 | hypothetical protein |
| Cluster_4912 | 10 | 10 | 0 | 9 | 10 | 4 | 0.0016 | 0.0411 | 0 | hypothetical protein |
| Cluster_4935 | 10 | 10 | 0 | 9 | 10 | 4 | 0.0016 | 0.0411 | 0 | hypothetical protein |
| Cluster_4982 | 10 | 10 | 0 | 9 | 10 | 4 | 0.0016 | 0.0411 | 0 | hypothetical protein |
| Cluster_4994 | 10 | 10 | 0 | 9 | 10 | 4 | 0.0016 | 0.0411 | 0 | hypothetical protein |
| Cluster_4997 | 10 | 10 | 0 | 9 | 10 | 4 | 0.0016 | 0.0411 | 0 | hypothetical protein |
| Cluster_4998 | 10 | 10 | 0 | 9 | 10 | 4 | 0.0016 | 0.0411 | 0 | hypothetical protein |
| Cluster_4890 | 10 | 10 | 0 | 9 | 10 | 4 | 0.0016 | 0.0411 | 0 | MerR family transcriptional regulator |
| Cluster_4987 | 10 | 10 | 0 | 9 | 10 | 4 | 0.0016 | 0.0411 | 0 | MFS transporter |
| Cluster_4868 | 10 | 10 | 0 | 9 | 10 | 4 | 0.0016 | 0.0411 | 0 | N-acetylmuramoyl-L-alanine amidase |
| Cluster_4855 | 10 | 10 | 0 | 9 | 10 | 4 | 0.0016 | 0.0411 | 0 | oligoendopeptidase F |
| Cluster_4834 | 10 | 10 | 0 | 9 | 10 | 4 | 0.0016 | 0.0411 | 0 | peptidase S8 |
| Cluster_4856 | 10 | 10 | 0 | 9 | 10 | 4 | 0.0016 | 0.0411 | 0 | peptide-binding protein |
| Cluster_4981 | 10 | 10 | 0 | 9 | 10 | 4 | 0.0016 | 0.0411 | 0 | RpiR family transcriptional regulator |
| Cluster_4908 | 10 | 10 | 0 | 9 | 10 | 4 | 0.0016 | 0.0411 | 0 | siderophore biosynthesis protein |
| Cluster_4895 | 10 | 10 | 0 | 9 | 10 | 4 | 0.0016 | 0.0411 | 0 | spore gernimation protein |
| Cluster_4599 | 11 | 10 | 0 | 9 | 10 | 4 | 0.0016 | 0.0411 | 0 | sporulation protein |
| Cluster_4600 | 11 | 10 | 0 | 9 | 10 | 4 | 0.0016 | 0.0411 | 0 | sporulation protein |
| Cluster_4897 | 10 | 10 | 0 | 9 | 10 | 4 | 0.0016 | 0.0411 | 0 | sugar kinase |
| Cluster_4857 | 10 | 10 | 0 | 9 | 10 | 4 | 0.0016 | 0.0411 | 0 | sulfonate ABC transporter ATP-binding protein |
| Cluster_4864 | 10 | 10 | 0 | 9 | 10 | 4 | 0.0016 | 0.0411 | 0 | transcriptional regulator |
| Cluster_4352 | 13 | 13 | 1 | 8 | 12 | 2 | 0.0007 | 0.0238 | 0.0276 | DNA-binding response regulator |
| Cluster_4312 | 13 | 13 | 1 | 8 | 12 | 2 | 0.0007 | 0.0238 | 0.0276 | general stress protein |
| Cluster_4308 | 13 | 13 | 1 | 8 | 12 | 2 | 0.0007 | 0.0238 | 0.0276 | hypothetical protein |
| Cluster_4332 | 13 | 13 | 1 | 8 | 12 | 2 | 0.0007 | 0.0238 | 0.0276 | hypothetical protein |
| Cluster_4342 | 13 | 13 | 1 | 8 | 12 | 2 | 0.0007 | 0.0238 | 0.0276 | hypothetical protein |
| Cluster_4353 | 13 | 13 | 1 | 8 | 12 | 2 | 0.0007 | 0.0238 | 0.0276 | hypothetical protein |
| Cluster_4351 | 13 | 13 | 1 | 8 | 12 | 2 | 0.0007 | 0.0238 | 0.0276 | two-component sensor histidine kinase |
| Cluster_4158 | 15 | 15 | 2 | 7 | 13 | 1 | 0.0010 | 0.0316 | 0.0291 | CAAX protease |

*^a^* P-values from two-sided Fisher's Exact Tests

*^b^* P-values were corrected using the False Discovery Rate (FDR)

*^c^* Odds ratios marked as INF (Infinite) are a result of dividing by zero
