# Supplementary material for: Genes Associated With Psychrotolerant Bacillus cereus Group Isolates
Source: Front Microbiol. 2019 Mar 29;10:662. doi: 10.3389/fmicb.2019.00662 (PMC6449464; doi:10.3389/fmicb.2019.00662)
Supplement: Supplementary file 5 [file Table_5.DOCX]

**Supplemental Table 5**: List of Gene Ontology terms significantly overrepresented in the genomes of psychrotolerant (> 1log_10_ increase after 21-day incubation at 6°C in BHI broth) *B. cereus* group isolates

| GO Terms | Presence Among Psychrot-olerant Strains | Absence Among Psychr-otolerant Strains | Presence Among Non-Psychrotolerant Strains | Absence Among Non-Psychrotolerant Strains | p-values | Odds Ratio | FDR-Corrected p-value | Description | GO Term Category | EC Annotation |
| --- | --- | --- | --- | --- | --- | --- | --- | --- | --- | --- |
| GO:0005886 | 5032 | 1340 | 7576 | 2336 | 1.55E-04 | 1.1578 | 0.0329 | plasma membrane | cellular component | NA^a^ |
| GO:0005524 | 4288 | 1535 | 6353 | 2705 | 3.92E-06 | 1.1894 | 0.0047 | ATP binding | molecular function | NA^a^ |
| GO:0016020 | 3193 | 1370 | 4724 | 2374 | 1.13E-04 | 1.1712 | 0.0299 | membrane | cellular component | NA^a^ |
| GO:0006810 | 1377 | 801 | 1966 | 1422 | 1.22E-04 | 1.2434 | 0.0299 | transport | biological process | NA^a^ |
| GO:0005622 | 1103 | 490 | 1515 | 963 | 1.39E-07 | 1.4307 | 0.0003 | intracellular | cellular component | NA^a^ |
| GO:0009365 | 628 | 272 | 858 | 542 | 3.23E-05 | 1.4582 | 0.0197 | protein histidine kinase complex | cellular component | NA^a^ |
| GO:0000155 | 612 | 270 | 832 | 540 | 2.35E-05 | 1.4709 | 0.0164 | phosphorelay sensor kinase activity | molecular function | NA^a^ |
| GO:0016616 | 147 | 69 | 174 | 162 | 1.98E-04 | 1.9811 | 0.0329 | oxidoreductase activity, acting on the CH-OH group of donors, NAD or NADP as acceptor | molecular function | NA^a^ |
| GO:0055088 | 86 | 4 | 106 | 34 | 4.18E-05 | 6.8499 | 0.0227 | lipid homeostasis | biological process | NA^a^ |
| GO:0009447 | 36 | 9 | 24 | 46 | 1.49E-06 | 7.5158 | 0.0024 | putrescine catabolic process | biological process | NA^a^ |
| EC:3.6.3.25 | 35 | 1 | 32 | 24 | 8.75E-06 | 25.55 | 0.0071 | sulfate-transporting ATPase | NA | NA^a^ |
| GO:0015419 | 35 | 1 | 32 | 24 | 8.75E-06 | 25.5509 | 0.0071 | ATPase-coupled sulfate transmembrane transporter activity | molecular function | NA^a^ |
| GO:0009263 | 27 | 0 | 26 | 16 | 9.64E-05 | NA^a^ | 0.0277 | deoxyribonucleotide biosynthetic process | biological process | NA^a^ |
| GO:0005768 | 26 | 10 | 18 | 38 | 2.45E-04 | 5.3769 | 0.0329 | endosome | cellular component | NA^a^ |
| EC:1.2.1.4 | 25 | 2 | 20 | 22 | 9.51E-05 | 13.2582 | 0.0277 | Aldehyde dehydrogenase (NADP(+)) | NA^a^ | NA^a^ |
| GO:0033721 | 25 | 2 | 20 | 22 | 9.51E-05 | 13.2582 | 0.0277 | aldehyde dehydrogenase (NADP+) activity | molecular function | NA^a^ |
| GO:0051063 | 18 | 0 | 12 | 16 | 6.02E-05 | NA^a^ | 0.0235 | CDP reductase activity | molecular function | NA^a^ |
| EC:3.1.1.31 | 9 | 0 | 2 | 12 | 6.73E-05 | NA^a^ | 0.0235 | 6-phospho  gluconolactonase. | NA^a^ | NA^a^ |
| GO:0008655 | 9 | 0 | 2 | 12 | 6.73E-05 | NA^a^ | 0.0235 | pyrimidine-containing compound salvage | biological process | NA^a^ |
| GO:0009174 | 9 | 0 | 2 | 12 | 6.73E-05 | NA^a^ | 0.0235 | pyrimidine ribonucleoside monophosphate biosynthetic process | biological process | NA^a^ |
| GO:0017057 | 9 | 0 | 2 | 12 | 6.73E-05 | NA^a^ | 0.0235 | 6-phosphogluconolactonase activity | molecular function | NA^a^ |
| EC:2.6.1.82 | 9 | 0 | 3 | 11 | 3.37E-04 | NA^a^ | 0.0329 | Putrescine aminotransferase | NA^a^ | PAT. Putrescine transaminase Putrescine-alpha-ketoglutarate transaminase |
| EC:3.7.1.14 | 9 | 0 | 3 | 11 | 3.37E-04 | NA^a^ | 0.0329 | 2-hydroxy-6-oxonona-2,4-dienedioate hydrolase | NA^a^ | NA^a^ |
| GO:0006082 | 9 | 0 | 3 | 11 | 3.37E-04 | NA^a^ | 0.0329 | organic acid metabolic process | biological process | NA^a^ |
| GO:0009258 | 9 | 0 | 3 | 11 | 3.37E-04 | NA^a^ | 0.0329 | 10-formyltetrahydrofolate catabolic process | biological process | NA^a^ |
| GO:0015489 | 9 | 0 | 3 | 11 | 3.37E-04 | NA^a^ | 0.0329 | putrescine transmembrane transporter activity | molecular function | NA^a^ |
| GO:0018771 | 9 | 0 | 3 | 11 | 3.37E-04 | NA^a^ | 0.0329 | 2-hydroxy-6-oxonona-2,4-dienedioate hydrolase activity | molecular function | NA^a^ |
| GO:0032383 | 9 | 0 | 3 | 11 | 3.37E-04 | NA^a^ | 0.0329 | regulation of intracellular cholesterol transport | biological process | NA^a^ |
| GO:0033094 | 9 | 0 | 3 | 11 | 3.37E-04 | NA^a^ | 0.0329 | butane-1,4-diamine:2-oxoglutarate aminotransferase activity | molecular function | NA^a^ |
| GO:0042632 | 9 | 0 | 3 | 11 | 3.37E-04 | NA^a^ | 0.0329 | cholesterol homeostasis | biological process | NA^a^ |
| GO:0048545 | 9 | 0 | 3 | 11 | 3.37E-04 | NA^a^ | 0.0329 | response to steroid hormone | biological process | NA^a^ |
| GO:0048870 | 9 | 0 | 3 | 11 | 3.37E-04 | NA^a^ | 0.0329 | cell motility | biological process | NA^a^ |
| GO:0052823 | 9 | 0 | 3 | 11 | 3.37E-04 | NA^a^ | 0.0329 | 2-hydroxy-6-oxonona-2,4,7-trienedioate hydrolase activity | molecular function | NA^a^ |
| GO:0055081 | 9 | 0 | 3 | 11 | 3.37E-04 | NA^a^ | 0.0329 | anion homeostasis | biological process | NA^a^ |

^a^ NA: Not Applicable
