# Supplementary material for: Genes Associated With Psychrotolerant Bacillus cereus Group Isolates
Source: Front Microbiol. 2019 Mar 29;10:662. doi: 10.3389/fmicb.2019.00662 (PMC6449464; doi:10.3389/fmicb.2019.00662)
Supplement: Supplementary file 6 [file Table_6.DOCX]

**Supplemental Table 6**: List of Gene Ontology terms significantly overrepresented in the genomes of non-psychrotolerant (< 1log_10_ increase after 21-day incubation at 6°C in BHI broth) *B. cereus* group isolates

| GO Terms | Presence Among Psychrot-olerant Strains | Absence Among Psychr-otolerant Strains | Presence Among Non-Psychrotolerant Strains | Absence Among Non-Psychrotolerant Strains | p-values | Odds Ratio | FDR Corrected  p-value | Description | GO Term Category | EC Annotation |
| --- | --- | --- | --- | --- | --- | --- | --- | --- | --- | --- |
| GO:0042959 | 0 | 18 | 22 | 6 | 5.03E-08 | 0 | 0.0002 | alkanesulfonate transporter activity | molecular function | NA |
| EC:1.14.14.5 | 0 | 9 | 11 | 3 | 3.37E-04 | 0 | 0.0329 | alkanesulfonate monooxygenase | NA^a^ | FMNH(2)-dependent aliphatic sulfonate monooxygenase. Sulfate starvation-induced protein 6. |
| GO:0006084 | 0 | 9 | 11 | 3 | 3.37E-04 | 0 | 0.0329 | acetyl-CoA metabolic process | biological process | NA^a^ |
| GO:0008504 | 0 | 9 | 11 | 3 | 3.37E-04 | 0 | 0.0329 | monoamine transmembrane transporter activity | molecular function | NA^a^ |
| GO:0008726 | 0 | 9 | 11 | 3 | 3.37E-04 | 0 | 0.0329 | alkanesulfonate monooxygenase activity | molecular function | NA^a^ |
| GO:0015370 | 0 | 9 | 11 | 3 | 3.37E-04 | 0 | 0.0329 | solute:sodium symporter activity | molecular function | NA^a^ |
| GO:0015807 | 0 | 9 | 11 | 3 | 3.37E-04 | 0 | 0.0329 | L-amino acid transport | biological process | NA^a^ |
| GO:0019427 | 0 | 9 | 11 | 3 | 3.37E-04 | 0 | 0.0329 | acetyl-CoA biosynthetic process from acetate | biological process | NA^a^ |
| GO:0046306 | 0 | 9 | 11 | 3 | 3.37E-04 | 0 | 0.0329 | alkanesulfonate catabolic process | biological process | NA^a^ |
| GO:0051715 | 0 | 18 | 14 | 14 | 1.98E-04 | 0 | 0.0329 | cytolysis in other organism | biological process | NA^a^ |
| GO:0090101 | 0 | 9 | 11 | 3 | 3.37E-04 | 0 | 0.0329 | negative regulation of transmembrane receptor protein serine/threonine kinase signaling pathway | biological process | NA^a^ |
| GO:1901289 | 0 | 9 | 11 | 3 | 3.37E-04 | 0 | 0.0329 | succinyl-CoA catabolic process | biological process | NA^a^ |
| GO:0010038 | 1 | 26 | 19 | 23 | 1.22E-04 | 0.048 | 0.0299 | response to metal ion | biological process | NA^a^ |

^a^ NA: Not Applicable
