# Supplementary material for: Genes Associated With Psychrotolerant Bacillus cereus Group Isolates
Source: Front Microbiol. 2019 Mar 29;10:662. doi: 10.3389/fmicb.2019.00662 (PMC6449464; doi:10.3389/fmicb.2019.00662)
Supplement: Supplementary file 7 [file Table_7.docx]

| Isolate | Clade | Growth at 6°C in BHI | CspA^a^ | | CspB^b^ | CspC^b^ | CspD^c^ | | | |  | CspE^d^ | | CspM^e^ | CspN^f^ |
| --- | --- | --- | --- | --- | --- | --- | --- | --- | --- | --- | --- | --- | --- | --- | --- |
|  |  |  | Psychro | Meso |  |  | D1_IIa-c | D1_IV-I/M | D2_IV-I | D2_IV-M |  | CspE_Fiedoruk_ | CspE_Mayr_ |  |  |
| FSL H8-0534 | I | No | + |  | + | + |  | + |  | + |  | + | + |  |  |
| FSL J3-0013 | II | No |  | + | + | + | + |  |  | + |  | + | + |  |  |
| FSL K6-0069 | II | No |  | + | + | + | + |  |  | + |  | + | + |  |  |
| FSL M8-0091 | II | No |  | + |  | + | + |  |  | + |  | + | + |  |  |
| FSL W9-0169 | II | No |  | + |  | + | + |  |  | + |  | + | + |  |  |
| FSL M8-0117 | III | No |  | + |  | + |  | + | + |  |  | + | + |  |  |
| FSL W8-0050 | III | No |  | + |  | + |  | + | + |  |  | + | + |  | + |
| FSL W8-0483 | III | No |  | + |  | + |  | + | + |  |  | + | + |  |  |
| FSL K6-1030 | IV | No |  | + |  | + |  | + | + |  |  | + | + |  |  |
| FSL M8-0473 | IV | No |  | + |  | + |  | + | + |  |  | + | + |  |  |
| FSL R5-0811 | IV | No |  | + |  | + |  | + | + |  |  | + | + |  |  |
| FSL W8-0268 | IV | No |  | + |  | + |  | + | + |  |  | + | + |  |  |
| FSL E2-0214 | VI | Yes | + |  | + | + | + |  |  | + |  | + | + | + |  |
| FSL H7-0683 | VI | No | + |  | + | + | + |  |  | + |  | + | + |  |  |
| FSL H7-0926 | VI | No | + |  | + | + | + |  |  | + |  | + | + | + |  |
| FSL H8-0485 | VI | Yes | + |  | + | + | + |  |  | + |  | + | + | + |  |
| FSL H8-0492 | VI | Yes | + |  | + | + | + |  |  | + |  | + |  |  |  |
| FSL J3-0123 | VI | Yes | + |  | + | + | + |  |  | + |  | + | + | + |  |
| FSL M7-0109 | VI | Yes | + |  | + | + | + |  |  | + |  | + | + | + |  |
| FSL M7-0609 | VI | Yes | + |  | + | + | + |  |  | + |  | + | + |  |  |
| FSL M7-1219 | VI | Yes | + |  | + | + | + |  |  | + |  | + | + | + |  |
| FSL R5-0708 | VI | Yes | + |  | + | + | + |  |  | + |  | + | + | + |  |
| FSL W7-1108 | VI | Yes | + |  | + | + | + |  |  | + |  | + | + | + |  |

Supplemental Table 7: Presence/absence of cold shock proteins from BLAST analyses

^a^Refers to the CspA_psychrotolerant_ and CspA_mesophilic_ variants described in (Mayr et al., 1996)

^b^Sequences for CspB and CpsC from (Mayr et al., 1996)

^c^Sequences for CspD proteins are named in accordance with (Fiedoruk et al., 2017)

^d^CspE_Fiedoruk_ from (Fiedoruk et al., 2017) and CpsE_Mayr_ from (Mayr et al., 1996)

^e^CspM from this study (OrthoMCL cluster_5279)

^f^CspN from this study, identified through NCBI annotation pipeline

References:

Fiedoruk, K., Drewnowska, J.M., Daniluk, T., Leszczynska, K., Iwaniuk, P., and Swiecicka, I. (2017). Ribosomal background of the *Bacillus* *cereus* group thermotypes. *Sci Report* 7**,** 46430.

Mayr, B., Kaplan, T., Lechner, S., and Scherer, S. (1996). Identification and purification of a family of dimeric major cold shock protein homologs from the psychrotrophic *Bacillus* *cereus* WSBC 10201. *J Bacteriol* 178**,** 2916-2925.
